# Vidarabine, an anti-herpes agent, prevents occlusal-disharmony-induced cardiac dysfunction in mice

**DOI:** 10.1186/s12576-022-00826-4

**Published:** 2022-02-11

**Authors:** Yoshio Hayakawa, Kenji Suita, Yoshiki Ohnuki, Yasumasa Mototani, Misao Ishikawa, Aiko Ito, Megumi Nariyama, Akinaka Morii, Kenichi Kiyomoto, Michinori Tsunoda, Ichiro Matsuo, Hiroshi Kawahara, Satoshi Okumura

**Affiliations:** 1 Department of Physiology, Tsurumi University School of Dental Medicine, 2-1-3 Tsurumi, Tsurumi-ku, Yokohama, 230-8501 Japan; 2 Department of Dental Anesthesiology, Tsurumi University School of Dental Medicine, Yokohama, 230-8501 Japan; 3 Department of Oral Anatomy, Tsurumi University School of Dental Medicine, Yokohama, 230-8501 Japan; 4 Department of Orthodontics, Tsurumi University School of Dental Medicine, Yokohama, 230-8501 Japan; 5 Department of Pediatric Dentistry, Tsurumi University School of Dental Medicine, Yokohama, 236-8501 Japan; 6 Department of Periodontology, Tsurumi University School of Dental Medicine, Yokohama, 230-8501 Japan

**Keywords:** β-Adrenergic signaling, Occlusal disharmony, Adenylyl cyclase, Apoptosis, Fibrosis, Signal transduction

## Abstract

We recently reported a positive relationship between occlusal disharmony and cardiovascular disease via activation of β-adrenergic signaling in mice. Furthermore, inhibition of type 5 adenylyl cyclase (AC5), a major cardiac subtype in adults, protects the heart against oxidative stress. Here, we examined the role of AC5 in the development of occlusal-disharmony-induced cardiovascular disease in bite-opening (BO) mice, prepared by cementing a suitable appliance onto the mandibular incisor. We first examined the effects of BO treatment on cardiac function in mice treated or not treated for 2 weeks with vidarabine, which we previously identified as an inhibitor of cardiac AC. Cardiac function was significantly decreased in the BO group compared to the control group, but vidarabine ameliorated the dysfunction. Cardiac fibrosis, myocyte apoptosis and myocyte oxidative DNA damage were significantly increased in the BO group, but vidarabine blocked these changes. The BO-induced cardiac dysfunction was associated with increased phospholamban phosphorylation at threonine-17 and serine-16, as well as increased activation of the Ca^2+^-calmodulin-dependent protein kinase II/receptor-interacting protein 3 signaling pathway. These data suggest that AC5 inhibition with vidarabine might be a new therapeutic approach for the treatment of cardiovascular disease associated with occlusal disharmony.

**Supplementary Information:**

The online version contains supplementary material available at 10.1186/s12576-022-00826-4.

## Introduction

Oral health decrease with age, and this is a major risk factor for many diseases, including cardiovascular disease [1]. In addition, aging-related loss of teeth, poor periodontal status and low alveolar bone levels can lead to occlusal disharmony [1, 2]. Increased sympathetic nervous activity was recently recognized as a hallmark feature that links aging with increased cardiovascular risk [3], and it is also involved in the etiology of oral frailty [4–6].

Adenylyl cyclase (AC) is the target enzyme of β-adrenergic receptor (β-AR) signaling stimulation. At least 9 isoforms are known, 7 of which are expressed in the heart; the type 5 isoform (AC5) is a major adult cardiac isoform, while type 6 is a major fetal isoform [7–9]. We have developed a mouse model with knock-out of AC5 (AC5KO) [10] and we have also identified vidarabine as a cardiac AC inhibitor in mice [11]. Using these models, we found that genetic disruption and pharmacological inhibition of AC5 are associated with resistance to the development of heart failure [10, 12, 13] and increased longevity [13, 14].

We previously examined the effects of occlusal disharmony on cardiac remodeling (fibrosis and myocyte apoptosis), cardiac function and susceptibility to atrial fibrillation in bite-opening mice (BO), in which a 0.7-mm BO was introduced by cementing a suitable appliance onto the mandibular incisor. We found that BO-induced cardiac dysfunction and susceptibility to atrial fibrillation are ameliorated by co-treatment with propranolol, a non-selective β-blocker [15, 16]. These findings indicate that activation of β-AR signaling might play a part in the cardiac dysfunction induced by occlusal disharmony.

However, β-blockers have several critical side effects. The inhibition of sympathetic signaling reduces cardiac function [17, 18], and great caution is required in the use of β-blockers for the treatment of heart failure and arrhythmia in aged patients [18]. We therefore proposed the usefulness of AC isoform-specific therapy, and we showed that vidarabine, which we previously identified as an inhibitor of cardiac AC, prevented the development of post-myocardial infarction heart failure and catecholamine-induced arrhythmia without worsening cardiac dysfunction [19, 20]. Therefore, in this work we examined the effects of AC isoform-specific therapy with vidarabine on occlusal-disharmony-induced cardiac dysfunction.

**Fig. 1:**
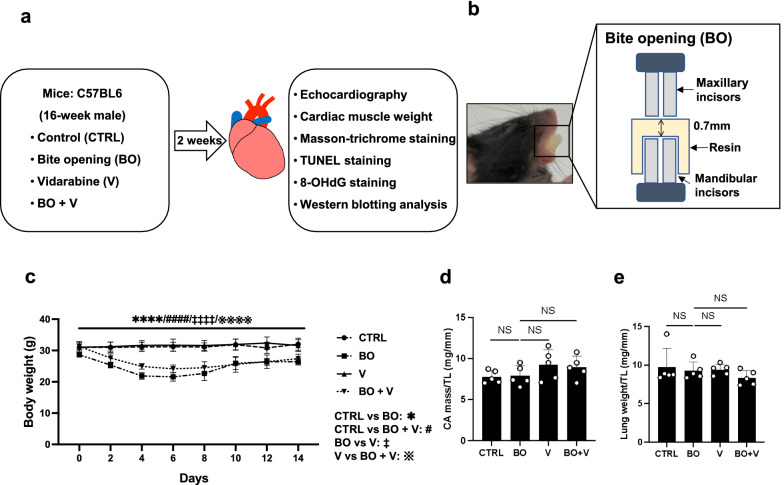
Schematic illustrations of experimental procedure and bite-opening treatment, and comparison of body weight, cardiac muscle weight and lung weight among the groups. **a** Male 16-week-old C57BL/6 mice were divided into four groups: a normal control group (CTRL), a bite-opening (BO)-treated group, a vidarabine-treated group (V), and a BO plus vidarabine-treated (BO + V) group. Long-term infusion of vidarabine was performed for 14 days at a dose of 15 mg/kg/day with the osmotic mini-pumps, and the indicated measurements were made. **b** Schematic representation of a bite-opening (BO) in the form of a 0.7 mm increase in the vertical height of occlusion, obtained by cementing a composite resin onto the mandibular incisors to cause occlusal disharmony in mice. **c** Body weight was measured daily for all animals throughout the 2-week experimental period. ****P < 0.0001 (Control (*n* = 5) vs. BO (*n* = 5), ^####^*P* < 0.0001 (Control vs. BO + V (*n* = 5), ^‡‡‡‡^*P* < 0.0001 (BO vs. V (*n* = 5)), ^※※※※^*P* < 0.0001 (V vs. BO + V) by two-way repeated-measures ANOVA followed by the Bonferroni post hoc test. **d**, **e** No significant difference in heart (**d**) or lung (**e**) size in terms of weight per tibial length ratio (mg/mm) at 2 weeks after BO treatment (Control (*n* = 5), BO (*n* = 5), vidarabine (*n* = 5) and BO + vidarabine (*n* = 5) groups; *P* = NS, not significantly different, vs. Control by one-way ANOVA) followed by the Tukey–Kramer post hoc test. Data are presented as mean ± SD

However, the role of AC5 in the occlusal-disharmony-induced cardiac deterioration remains poorly understood. In this study, therefore, we examined the effects of AC5 inhibition with vidarabine on cardiac function, cardiac fibrosis, myocyte apoptosis, and oxidative DNA damage induced by occlusal disharmony in mice (Fig. 1a, b, Additional file 1: Fig. S1). Acute and chronic treatment with vidarabine does not alter basal cardiac function in healthy animals, unlike β-blocker administration [20, 21]. More importantly, vidarabine has been used as an anti-viral drug for many years in humans [11, 20]. Therefore, vidarabine, rather than β-blocker, might be a safe and immediately clinically available drug for the treatment or prevention of cardiac dysfunction induced by occlusal disharmony.

## Materials and methods

### Mice and experimental protocols

All experiments were performed on male 12-week-old C57BL/6 mice obtained from CLEA Japan (Tokyo, Japan). Mice were group-housed at 23 °C under a 12–12 light/dark cycle with lights on at 8:00 AM in accordance with the standard conditions for mouse studies by our group [15, 16, 22, 23]. Both food and water were available ad libitum.

Occlusal disharmony in mice was induced by introducing a 0.7-mm BO, employing our standard method of cementing a suitable appliance onto the mandibular incisor under intraperitoneal anesthesia with medetomidine (0.03 mg/ml), midazolam (0.4 mg/ml), and butorphanol (0.5 mg/ml) [15, 16, 24]. Mice were divided into four groups: a normal control group (Control), a BO-only treatment group (BO), a vidarabine-only treatment group (V), and a BO plus vidarabine treatment group (BO + V) (Fig. 1a, b). Chronic infusion of vidarabine dissolved in DMSO (#359-13471; Sigma, St. Louis MO, USA) was performed for 14 days at a dose of 15 mg/kg/day delivered with osmotic mini-pumps (Model 2002; ALZET, Cupertino, CA, USA) [20, 25]. The dose of vidarabine (15 mg/kg/day) was selected based upon that used in previous studies: this dose did not eliminate the inotropic effects of acute isoproterenol, did not depress cardiac function at baseline, and retained high selectivity for AC5 [20]. Because the BO mice cannot easily eat the standard pellet food (CE-2: 334.9 kcal/100 g; CLEA Japan), but can take paste food, the standard pellet food was changed to paste food 3 days before the BO treatment in all groups, as in previous studies [24, 26]. Body weight (BW) (*n* = 5 each) (Fig. 1c), food intake (Control: *n* = 4, BO: *n* = 6, V: *n* = 4, BO + V: *n* = 6) (Additional file 1: Fig. S2a) and water intake (Control: *n* = 4, BO: *n* = 5, V: *n* = 5, BO + V: *n* = 6) (Additional file 1: Fig. S2b) were monitored throughout the 2-week experimental period.

### Physiological experiments

Mice (Control: *n* = 6, BO: *n* = 6, V: *n* = 7, BO + V: *n* = 6) were anesthetized via a mask with isoflurane vapor (1.0–1.5% v/v) titrated to maintain the lightest anesthesia possible, and echocardiographic measurements were performed by means of ultrasonography (TUS-A300, Toshiba, Tokyo, Japan) at 14 days after BO treatment [27].

### Evaluation of fibrosis

Among several quantitative methods available to determine interstitial fibrotic regions [23, 28, 29], we employed Masson-trichrome staining using the Accustatin Trichrome Stain Kit (#HT15-1KT; Sigma-Aldrich, St. Louis, MO, USA) in accordance with the manufacturer’s protocol, as described previously [15, 16, 22, 23]. Cross sections (10 μm) were cut with a cryostat (CM1900; Leica Microsystems, Nussloch, Germony). The sections were air-dried and fixed with 4% paraformaldehyde (v/v) in 0.1 M phosphate-buffered saline (pH 7.5) (Control: *n* = 4, BO: *n* = 5, V: *n* = 4, BO + V: *n* = 4) [15, 16, 22, 23]. We quantified interstitial fibrotic regions using freely available image analysis software (Image J 1.48; https://imagej.nih.gov/ij/download.html) to evaluate the percentage of blue area in the Masson-trichrome sections [15, 16, 22, 23].

### Evaluation of apoptosis

Apoptosis was determined by terminal deoxyribonucleotidyl transferase (TdT)-mediated biotin-16-deoxyuridine (TUNEL) staining using an Apoptosis in situ Detection Kit (#293-71501; Wako, Osaka, Japan). TUNEL-positive nuclei per field of view were manually counted in six sections of each of the four groups (Control: *n* = 4, BO: *n* = 5, V: *n* = 5, BO + V: *n* = 4) over a microscopic field of 20 x, then averaged and expressed as the ratio of TUNEL-positive nuclei (%) [27, 30]. By limiting the counting of total nuclei and TUNEL-positive nuclei to areas containing true cross sections of myocytes, we could selectively count only those nuclei that were clearly located within myocytes.

### Western blotting

Cardiac muscle excised from mice (Control: *n* = 10, BO: *n* = 8, V: *n* = 6, BO + V: *n* = 8) was homogenized in a Polytron (Kinematica AG, Lucerne, Switzerland) in ice-cold RIPA buffer (Thermo Fisher Scientific, Waltham, MA, USA: 25 mM Tris–HCl (pH 7.6), 150 mM NaCl, 1% NP-40, 1% sodium deoxycholate, 0.1% SDS) without addition of inhibitors [31], and the homogenate was centrifuged at 13,000 × *g* for 10 min at 4 °C. The supernatant was collected and the protein concentration was measured using a DC protein assay kit (Bio-Rad, Hercules, CA, USA). Equal amounts of protein (5 μg) were subjected to 12.5% SDS–polyacrylamide gel electrophoresis and blotted onto 0.2 mm PVDF membrane (Millipore, Billerica, MA, USA).

**Fig. 2:**
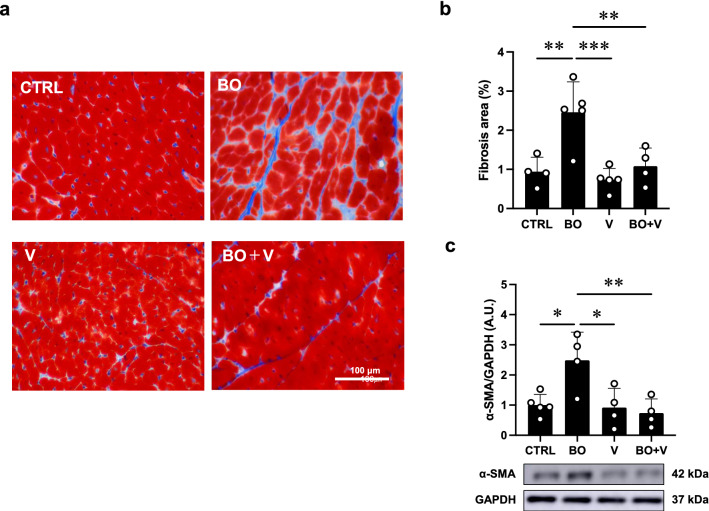
Effect of vidarabine on BO-induced fibrosis in the heart. **a** Representative images of Masson-trichrome-stained sections of cardiac muscle in the Control (CTRL) (upper left), BO (upper right), vidarabine (V) (lower left) and BO + V; lower right) groups. **b** The area of fibrosis was significantly increased in the BO group (*n* = 5), but this increase was blocked in the BO + V group (*n* = 4). ^**^P < 0.01, ^***^P < 0.001 by one-way repeated-measures ANOVA followed by the Tukey–Kramer post hoc test. **c** Expression of α-SMA, a fibrosis-related gene, was significantly increased in the BO group (*n* = 4), but this increase was blocked in the BO + V group (*n* = 4). ^*^P < 0.05, ^**^P < 0.01 by one-way ANOVA followed by the Tukey–Kramer post hoc test. Data are presented as mean ± SD. Full-size images of immunoblots are presented in Additional file 1: Fig. S3

**Fig. 3:**
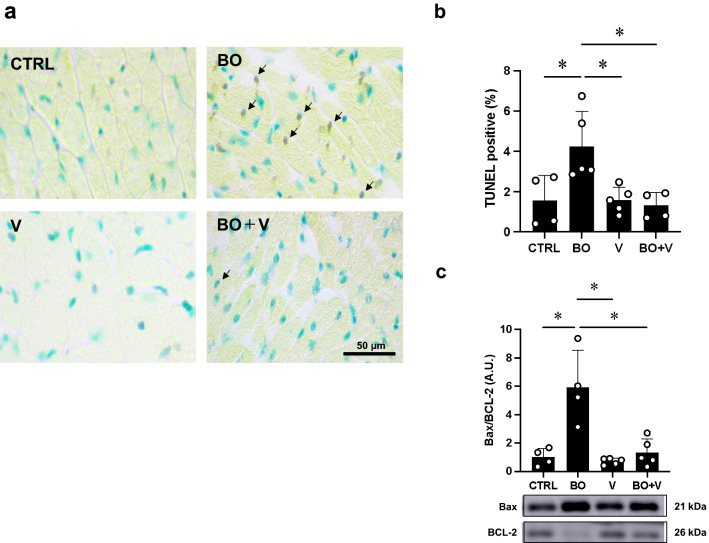
Effect of vidarabine on BO-induced cardiac myocyte apoptosis. **a** TUNEL-positive nuclei (black arrows) in representative TUNEL-stained sections were counted in cardiac muscle in the Control (CTRL; upper left), BO (upper right), Vidarabine (V; lower left) and BO + V (lower right) groups. **b** The number of TUNEL-positive nuclei was significantly increased in the BO group (*n* = 5), but this increase was blocked in the BO + V group (*n* = 4). ^*^P < 0.05 by one-way ANOVA followed by the Tukey–Kramer post hoc test. **c** The Bax/BCL-2 ratio was significantly increased in the BO group (*n* = 4), but this increase was blocked in the BO + V group (*n* = 5). ^*^P < 0.05 by one-way ANOVA followed by the Tukey–Kramer post hoc test. Data are presented as mean ± SD. Full-size images of immunoblots are presented in Additional file 1: Fig. S4

**Fig. 4:**
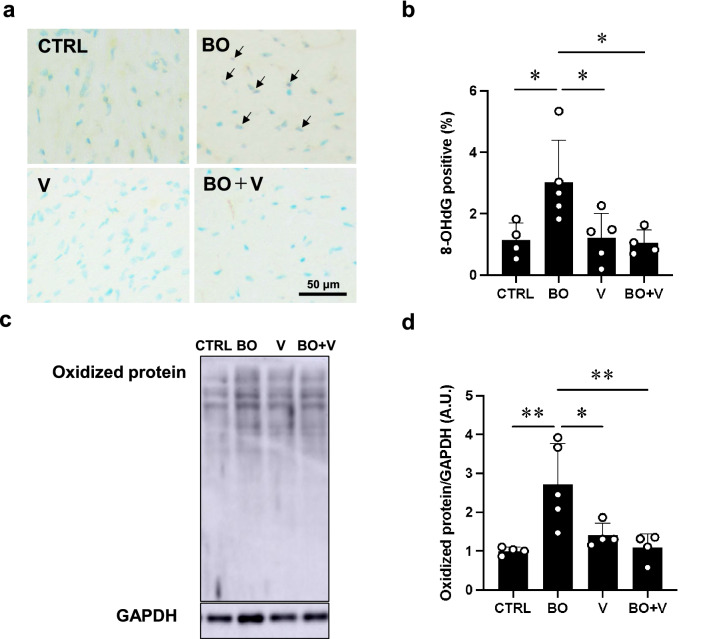
Effect of vidarabine on BO-induced oxidative stress in cardiac muscle. **a** Representative immunohistochemical images of oxidative DNA damage (8-OHdG) in cardiac muscle in the Control (CTRL; upper left), BO (upper right), vidarabine (V; lower left) and BO + V (lower right) groups. **b** 8-OHdG-positive nuclei were significantly increased in the BO group (*n* = 5), but this increase was blocked in the BO + V group (*n* = 4). ^*^P < 0.05 by one-way ANOVA followed by the Tukey–Kramer post hoc test. **c** Representative SDS-PAGE of oxidized proteins in cardiac muscle homogenate prepared from Control (CTRL; lane 1), BO (lane 2), V (lane 3) and BO + V (lane 4) groups using the OxiSelect^TM^ Protein Carbonyl Immunoblot Kit. Full-size images of immunoblots are presented in Additional file 1: Fig. S6. **d** Oxidized proteins were significantly increased in the BO group (*n* = 5), but this increase was blocked in the BO + V group (*n* = 4). ^*^P < 0.05, ^**^P < 0.01 by one-way ANOVA followed by the Tukey–Kramer post hoc test. Data are presented as mean ± SD

**Fig. 5:**
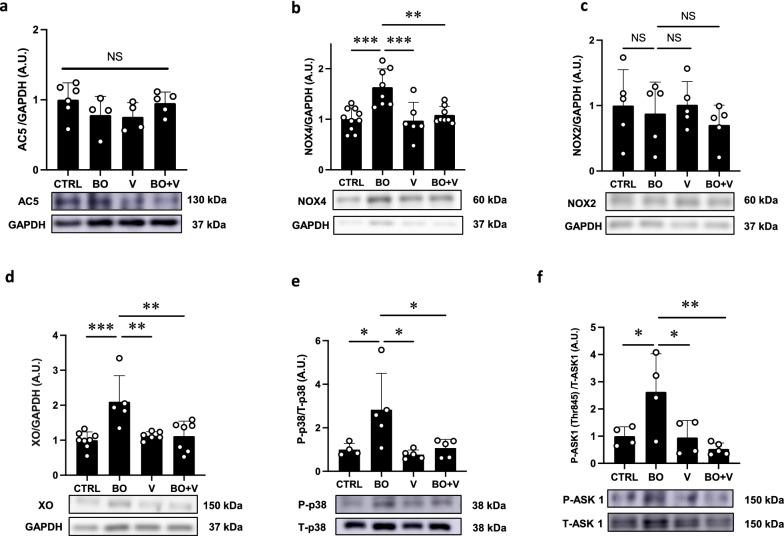
Effect of vidarabine on AC5, NOX4/2, XO, phospho-p38 and phospho-ASK1 in the heart of BO mice. **a** AC5 expression was similar in all four groups. NS, not significantly different, by one-way ANOVA followed by the Tukey–Kramer post hoc test. Full-size images of immunoblots are presented in Additional file 1: Fig. S7. **b** NOX4 expression was significantly increased in the BO group (*n* = 8), and this increase was significantly blocked in the BO + V group (*n* = 8). ^**^P < 0.01, ^***^P < 0.001 by one-way ANOVA followed by the Tukey–Kramer post hoc test. Full-size images of immunoblots are presented in Additional file 1: Fig. S8. **c** NOX2 expression was similar among the four groups (*n* = 5 each). NS, not significantly different, by one-way ANOVA followed by the Tukey–Kramer post hoc test. Full-size images of immunoblots are presented in Additional file 1: Fig. S9.** d** Expression of XO was significantly increased in the BO group (*n* = 5), and this increase was significantly blocked in the BO + V group (*n* = 7). ^**^P < 0.01, ^***^P < 0.001, ^***^P < 0.001 by one-way ANOVA followed by the Tukey–Kramer post hoc test. Full-size images of immunoblots are presented in Additional file 1: Fig. S10. **e** Expression of phospho-p38 was significantly increased in the BO group (*n* = 4) and this was significantly blocked in the BO + V group (*n* = 5). ^*^P < 0.05 by one-way ANOVA followed by the Tukey–Kramer post hoc test. Full-size images of immunoblots are presented in Additional file 1: Fig. S11. **f** Expression of phospho-ASK1 (Thr-845) was significantly increased in the BO group (*n* = 4) and this increase was significantly blocked in the BO + V group (*n* = 5). ^*^P < 0.05 by one-way ANOVA followed by the Tukey–Kramer post hoc test. Full-size images of immunoblots are presented in Additional file 1: Fig. S12. Data are presented as mean ± SD

**Fig. 6:**
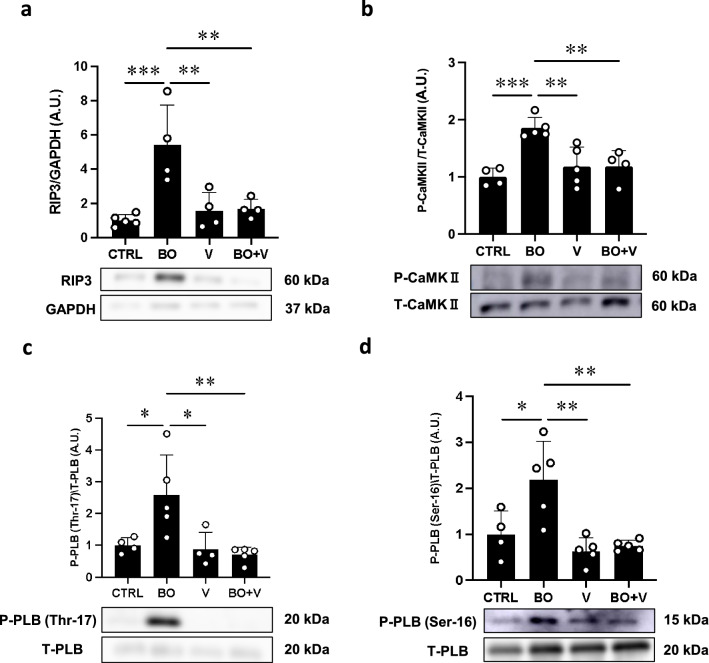
Effect of vidarabine on BO-induced RIP3, phospho-CaMKII and phospho-PLN in the heart of BO mice. **a** Expression of RIP3, a key mediator of necroptosis, was significantly increased in the BO group (*n* = 4), but this increase was significantly blocked in the BO + V group (*n* = 4). ^**^P < 0.01, ^***^P < 0.001 by one-way ANOVA followed by the Tukey–Kramer post hoc test. Full-size images of immunoblots are presented in Additional file 1: Fig. S13. **b** Expression of phospho-CaMKII (Thr-286) was significantly increased in the BO group (*n* = 5), but this increase was significantly blocked in the BO + V group (*n* = 4). ^*^P < 0.05, ^**^P < 0.01, ^***^P < 0.001 by one-way ANOVA followed by the Tukey–Kramer post hoc test. Full-size images of immunoblots are presented in Additional file 1: Fig. S14.** c** Expression of phospho-PLN (Thr-17) was significantly increased in the BO group (*n* = 5), but this increase was blocked in the BO + V group (*n* = 4). ^*^P < 0.05, ^**^P < 0.01 by one-way ANOVA followed by the Tukey–Kramer post hoc test. Full-size images of immunoblots are presented in Additional file 1: Fig. S15. **d** Expression of phospho-PLN (Ser-16) was significantly increased in the BO group (*n* = 5), but this increase was significantly blocked in the BO + V group (*n* = 5). ^*^P < 0.05, ^**^P < 0.01 by one-way ANOVA followed by the Tukey–Kramer post hoc test. Full-size images of immunoblots are presented in Additional file 1: Fig. S16. Data are presented as expressed as mean ± SD

Western blotting was conducted with commercially available antibodies [10, 27, 30]. Primary antibodies against α-smooth muscle actin (α-SMA) (1:1000, #19245), calmodulin kinase II (CaMKII) (1:1000, #3362), phospho-CaMKII (1:1000, Thr-286, #3361), B cell lymphoma 2 (BCL-2) (1:1000, #3498), BCL-2 associated X (Bax) (1:1000, #2772), receptor-interacting protein 3 (RIP3) (1:1000, #95702), phospho-RIP3 (1:1000, Thr-231/Ser-232, #91702), p38 (1:1000, #8690), phospho-p38 (1:1000, Thr-180/Tyr-182, #4511), apoptosis signal-regulatory kinase 1 (ASK1) (1:1000, #8662) and phospho-ASK1 (1:1000, Thr-845, #3765) were purchased from Cell Signaling Technology (Boston, MA, USA), primary antibodies against glyceraldehyde-3-phosphate dehydrogenase (GAPDH) (1:200, sc-25778) were purchased from Santa Cruz Biotechnology (Santa Cruz, CA, USA) and primary antibodies against phospho-phospholamban (PLN) (1:5000, Thr-17, #A010-13; 1:5000, Ser-16, #A010-12) and PLN (1:5000, #A010-14) were purchased from Badrilla (Leeds, UK). Primary antibodies against nicotinamide adenine dinucleotide phosphate oxidase (NOX) 4 (1:1000, #ab133303), NOX2 (1:1000, #ab80508) and xanthine oxidase (XO) (1:1000, #ab109235) were purchased from Abcam (Cambridge, UK) and AC5 (1:1000, #SAB4500206) were purchased from Sigma. Horseradish peroxide-conjugated anti-rabbit (1:5000, #NA934) or anti-mouse IgG (1:5000, #NA931) purchased from GB Healthcare was used as the secondary antibody. The primary and secondary antibodies were diluted in Tris-buffered saline (pH 7.6) with 0.1% Tween 20 and 5% bovine serum albumin. The blots were visualized with enhanced chemiluminescence solution (ECL: Prime Western Blotting Detection Reagent, GE Healthcare, Piscataway, NJ, USA) and scanned with a densitometer (LAS-1000, Fuji Photo Film, Tokyo, Japan). Note that there are different numbers of samples in different western blotting figures (Figs. 2, 3, 4, 5, 6) because we excluded outliers (extremely low or high values compared to others in the same groups).

### Immunostaining

Oxidative DNA damage in the myocardium was evaluated by immunostaining for 8-hydroxy-2’-deoxyguanosine (8-OHdG) using the Vector M.O.M Immunodetection system (Control: *n* = 4, BO: *n* = 5, V: *n* = 5, BO + V: *n* = 4) (#PK-2200, Vector Laboratories, Inc. Burlingame, CA, USA) under our standard conditions [15, 16]. Cross sections were cut at 10 μm with a cryostat at − 20 ºC, air-dried and fixed with 4% paraformaldehyde (v/v) in TBS-T for 5 min at room temperature. Antigen retrieval was achieved with 0.1% citrate plus 1% Triton X-100 for 30 min at room temperature, then the sections were washed with TBS-T, incubated with 0.3% horse serum in TBS-T for 1 h at room temperature, and blocked with M.O.M. blocking reagent (Vector Laboratories, Burlingame, CA, USA) overnight at 4 ºC. For the positive control, sections were incubated with 0.3% H_2_O_2_ in TBS-T before the anti-8-OHdG antibody treatment. The sections were incubated with anti-8-OHdG antibody (8.3 μg/ml in M.O.M. Dilute; clone N45.1 monoclonal antibody; Japan Institute for the Control of Aging, Shizuoka, Japan) overnight at 4ºC in a humidified chamber, then incubated with 0.3% H_2_O_2_ in 0.3% horse serum for 1 h at room temperature to inactivate endogenous peroxidase, rinsed with TBS-T, incubated with anti-mouse IgG in M.O.M. Diluent, and processed with an ABC kit (Vector Laboratories, Inc. Burlingame, CA, USA). The ratio of 8-OHdG nuclei with oxidative DNA damage (stained dark brown) per total cell number was evaluated.

### Statistical analysis

Data are presented as means ± standard deviation (SD). Comparisons were performed using two-way repeated-measures ANOVA followed by the Bonferroni post hoc test (Fig. 1c, Additional file 1: Fig. S2a, b) or one-way ANOVA followed by Tukey’s post hoc test (Figs. 1d, e, 2b, c, 3b, c, 4b, d, 5a–f, 6a–d). Differences were considered significant when *P* < 0.05.

## Results

### Effects of BO on body weight with/without vidarabine co-treatment

The BW in the control and vidarabine groups showed no significant change during the experimental period (Fig. 1c). However, BW gradually decreased in the BO and BO plus vidarabine treatment groups (BO + vidarabine), reaching a minimum at 4–6 days after the BO treatment, in accordance with previous findings by our group [15] and others [24, 32]. After that, the BW of the BO and BO + vidarabine groups gradually increased, but did not reach the preoperative level during the experimental period (Fig. 1c).

### Effects of BO on the consumption of food and drinking water

We monitored the daily consumption of pellet food and water per mouse, measured as an average of the animals in each cage, during the 2-week experimental period. Consumption levels of food (Additional file 1: Fig. S2a) and water (Additional file 1: Fig. S2b) in the Control and vidarabine groups were similar and did not show significant changes during the period. The BO and BO + vidarabine groups might have some difficulty eating and the consumption of food and water was minimum at 1 day after the BO treatment. However, consumption recovered to preoperative levels within 6 days and no significant difference was observed among the four groups at 2 weeks. Changes in the consumption of water showed a similar tendency to those of food (Additional file 1: Fig. S2b).

### Effects of BO on heart size and lung weight with/without vidarabine co-treatment

We examined the effect of BO with/without vidarabine on heart size in terms of cardiac muscle mass per tibial length ratio (Fig. 1d) and the effect on wet lung weight in terms of wet lung weight per tibial length ratio (Fig. 1e). Similar results were obtained among the four groups (*n* = 5 each).

These data suggest that BO did not induce cardiac hypertrophy or lung edema during the experimental period.

### Vidarabine inhibits BO-induced cardiac fibrosis

We examined cardiac fibrosis in BO mice with/without vidarabine by means of Masson-trichrome staining (Fig. 2a, b). BO treatment significantly increased the area of fibrosis in cardiac muscle (Control (*n* = 4) vs. BO (*n* = 5); 0.9 ± 0.4% vs. 2.5 ± 0.8%, *P* < 0.01 vs. Control) in accordance with our previous finding [15]. Vidarabine alone did not alter the area of fibrosis, but it blocked the BO-induced increase of fibrosis (BO (*n* = 5) vs. BO + vidarabine (*n* = 4); 2.5 ± 0.8% vs. 1.1 ± 0.5%, *P* < 0.001 vs. BO).

We also evaluated cardiac fibrosis by measuring the level of α-SMA expression at 2 weeks after the start of BO, because this parameter is closely associated with cardiac fibrosis [33]. Expression of α-SMA was significantly increased in cardiac muscle of BO mice (Control (*n* = 5) vs. BO (*n* = 4); 1.0 ± 0.4 vs. 2.5 ± 0.9 arbitrary unit (A.U.), *P* < 0.05 vs. Control), and the increase was significantly suppressed by vidarabine (BO (*n* = 4) vs. BO + vidarabine (*n* = 4); 2.5 ± 0.9 vs. 0.7 ± 0.5 A.U., *P* < 0.01 vs. BO) (Fig. 2c).

These data, together with our previous findings [20], suggest that cardiac fibrosis induced by BO might be mediated at least in part through the activation of AC5.

### Vidarabine inhibits BO-induced cardiac myocyte apoptosis

We also examined cardiac myocyte apoptosis in BO mice with/without vidarabine by means of TUNEL staining (Fig. 3a, b). BO treatment significantly increased cardiac myocyte apoptosis (Control (*n* = 4) vs. BO (*n* = 5); 1.6 ± 1.3% vs. 4.2 ± 1.7%, *P* < 0.05 vs. Control) in accordance with our previous findings [15]. Vidarabine alone (*n* = 5) had no effects on the number of TUNEL-positive cardiac myocytes, but it blocked the BO-induced increase of TUNEL-positive cardiac myocytes (BO (*n* = 5) vs. BO + vidarabine (*n* = 4); 4.2 ± 1.7% vs. 1.3 ± 0.7%, *P* < 0.05 vs. Control).

We also evaluated cardiac myocyte apoptosis by examining the ratio of Bax protein, an accelerator of apoptosis, and BCL-2 protein, a regulator of apoptosis, in the heart (Fig. 3c).

BO treatment significantly increased the ratio of Bax/BCL-2 in the heart (Control (*n* = 4) vs. BO (*n* = 4); 1.0 ± 0.6% vs. 5.9 ± 2.6 A.U., *P* < 0.05 vs. Control). Vidarabine alone (*n* = 5) had no effects on the ratio of Bax/BCL-2, but it blocked the BO-induced increase in the Bax/BCL-2 ratio (BO (*n* = 4) vs. BO + vidarabine (*n* = 5); 5.9 ± 2.6 vs. 1.3 ± 1.0 A.U., *P* < 0.05 vs. Control) (Fig. 3c).

These and earlier results [11] support the idea that the increase of TUNEL-positive cardiac myocytes induced by BO treatment might be mediated at least in part through the activation of AC5.

### Vidarabine inhibits BO-induced cardiac dysfunction

We performed echocardiography to evaluate cardiac function in terms of left ventricular cardiac function (EF) and fractional shortening (%FS) (Table 1). Both parameters were significantly decreased in the BO group compared to the control mice (EF: Control (*n* = 6) vs. BO (*n* = 6); 68 ± 1.1% vs. 61 ± 3.0%, *P* < 0.001 vs. Control; %FS: Control (*n* = 6) vs. BO (*n* = 6); 33 ± 0.8% vs. 28 ± 1.9%, *P* < 0.001 vs. Control). Vidarabine alone (*n* = 7) had no effect on EF or %FS, but blocked the BO-induced decrease of EF and %FS (EF: BO (*n* = 6) vs. BO + vidarabine (*n* = 6); 61 ± 3.0% vs. 66 ± 1.4%, *P* < 0.01 vs. Control; %FS: BO (*n* = 6) vs. BO + vidarabine (*n* = 6); 28 ± 1.9% vs. 31 ± 1.0%, *P* < 0.01 vs. Control).

**Table 1:** Cardiac function assessed by echocardiography at 2 weeks after BO

	Control	BO	*V*	BO + *V*
*n*	6	6	7	6
IVSTd	0.57 ± 0.06	0.53 ± 0.08	0.59 ± 0.08	0.51 ± 0.05
LVSTs	0.95 ± 0.07	0.86 ± 0.03	0.92 ± 0.07	0.84 ± 0.04^*^
LVEDV	0.21 ± 0.03	0.21 ± 0.01	0.21 ± 0.03	0.19 ± 0.03
CO	57 ± 8.6	54 ± 4.5	60 ± 8.9	48 ± 9.6
HR	468 ± 57	468 ± 20	473 ± 62	434 ± 73
LVIDd	4.4 ± 0.2	4.4 ± 0.1	4.4 ± 0.3	4.2 ± 0.2
LVIDs	3.0 ± 0.1	3.2 ± 0.1	2.9 ± 0.4	2.9 ± 0.2
ESV	0.07 ± 0.009	0.08 ± 0.009	0.07 ± 0.02	0.06 ± 0.01^#^
EF	68 ± 1.1	61 ± 3.0^***^	67 ± 2.3	66 ± 1.4^##^
LVPWTd	0.56 ± 0.03	0.54 ± 0.04	0.59 ± 0.04	0.56 ± 0.04
LVPWTs	0.91 ± 0.06	0.86 ± 0.06	0.91 ± 0.07	0.84 ± 0.09
SV	0.14 ± 0.02	0.13 ± 0.01	0.14 ± 0.02	0.12 ± 0.02
%FS	33 ± 0.8	28 ± 1.9^***^	32 ± 1.5	31 ± 1.0^##^

IVSTd (mm): interventricular septum thickness at end-diastole, LVSTs (mm): interventricular septum thickness at end-systole, LVEDV (mL): left ventricular end-diastolic volume, CO (mL/min): cardiac output, LVIDd (mm): left ventricular internal dimension at end-diastole, LVIDs (mm): left ventricular internal dimension at end-diastole, ESV (mL): left ventricular end-systolic volume, EF (%): ejection fraction, LVPWTd (mm): left ventricular posterior wall thickness at end-diastole, LVPWTs (mm): left ventricular posterior wall thickness at end-diastole, SV (mL): stroke volume, %FS (%): % fractional shortening

***P < 0.001 vs. Control

^##^*P* < 0.05 vs. BO

^#^*P* < 0.05 vs. BO

These data suggest that BO treatment decreases cardiac function at least in part through the activation of AC5.

### Vidarabine inhibits BO-induced oxidative stress

We evaluated oxidative stress in the myocardium by means of 8-OHdG immunostaining (Fig. 4a, b) and western blotting of oxidized proteins (Fig. 4c, d).

We first prepared positive and negative control sections by incubating cells with (positive control)/without (negative control) 0.3% H_2_O_2_ in TBS-T for 1 h at room temperature before the anti-8-OHdG antibody treatment and confirmed that the 8-OHdG staining procedure could clearly discriminate 8-OHdG-positive and non-positive nuclei (Additional file 1: Fig. S5).

The ratio of 8-OHdG-positive/total cells was significantly increased in the BO group (Control (*n* = 4) vs. BO (*n* = 5); 1.1 ± 0.6% vs. 3.0 ± 1.4%, *P* < 0.05 vs. Control), and the increase was suppressed by vidarabine (BO (*n* = 5) vs. BO + vidarabine (*n* = 4); 3.0 ± 1.4% vs. 1.1 ± 0.4%, *P* < 0.05 vs. Control).

The amount of oxidized proteins, measured using the OxiSelect^TM^ protein kit, was also significantly increased (Control (*n* = 4) vs. BO (*n* = 5); 1.0 ± 0.1 vs. 2.7 ± 1.0 A.U., *P* < 0.01 vs. Control), and again the increase was suppressed by vidarabine (BO (*n* = 5) vs. BO + vidarabine (*n* = 4); 2.7 ± 1.0 vs. 1.1 ± 0.4 A.U., *P* < 0.05 vs. Control).

These results, together with the data shown in Figs. 2, 3 and Table 1, indicate that BO treatment increases oxidative stress-induced myocardial damage at least in part through the activation of AC5, which might contribute to the cardiac remodeling and dysfunction in BO mice.

### Effects of BO on AC5 expression

We first examined the effects of BO treatment on AC5 expression with/without vidarabine, since AC5 was previously found to be increased by chronic catecholamine stress [12]. However, BO treatment did not alter AC5 expression and similar results were obtained in all four groups (Fig. 5a).

### Vidarabine inhibits BO-mediated NOX4 and XO expression

Reactive oxygen species (ROS) are produced through a number of pathways, including NOX and XO, and may be involved in various physiological and pathological processes in the heart, including fibrosis, apoptosis and heart failure [34–37].

Two NOX isoforms, NOX2 and NOX4, are expressed in the heart, and their activity is regulated by their expression level [35, 38]. We therefore examined NOX4 and NOX2 protein expression in the heart among the four groups. NOX4 expression was significantly increased in the BO group (Control (*n* = 10) vs. BO (*n* = 8); 1.0 ± 0.2 vs. 1.6 ± 0.4 A.U., *P* < 0.001 vs. Control). This increase was significantly inhibited by vidarabine (BO (*n* = 8) vs. BO + vidarabine (*n* = 8); 1.6 ± 0.4 vs. 1.1 ± 0.2 A.U., *P* < 0.01 vs. BO) (Fig. 5b). We also examined NOX2 protein expression and found that it was similar among the four groups (*n* = 5 each) (Fig. 5c).

We next examined XO expression in the heart in the four groups. XO expression was significantly increased in the BO group (Control (*n* = 8) vs. BO (*n* = 5); 1.0 ± 0.2 vs. 2.1 ± 0.7 A.U., *P* < 0.001 vs. Control). This increase was significantly inhibited by vidarabine (BO (*n* = 5) vs. BO + vidarabine (*n* = 7); 2.1 ± 0.7 vs. 1.1 ± 0.4 A.U., *P* < 0.01 vs. BO) (Fig. 5d).

These data suggest that activation of AC5 might contribute, at least in part, to the upregulation of NOX4 and XO.

### Vidarabine inhibits BO-mediated activation of p38 MAPK

Overproduction of ROS derived from NOX4 and XO triggers oxidative stress and subsequently activates the p38 mitogen-activated protein kinase (MAPK) signaling pathway, leading to cardiac dysfunction [38–40]. We therefore examined the phosphorylation levels of p38 MAPK and found that p38 MAPK phosphorylation was significantly increased in the BO group (Control (*n* = 4) vs. BO (*n* = 5); 1.0 ± 0.3 vs. 2.8 ± 1.7 A.U., *P* < 0.05 vs. Control). This increase was significantly inhibited by vidarabine (BO (*n* = 5) vs. BO + vidarabine (*n* = 5); 2.8 ± 1.7 vs. 1.1 ± 0.4 A.U., *P* < 0.05 vs. Control) (Fig. 5e).

These data suggest that overproduction of ROS derived from BO-induced upregulation of NOX4 and XO activates the p38 MAPK signaling pathway, which might lead to cardiac remodeling and dysfunction.

### Vidarabine inhibits BO-mediated activation of ASK1

ROS production was recently demonstrated to increase activation of ASK1, which signals selectively to p38 MAPK and orchestrates cardiac remodeling in response to pressure overload, chronic catecholamine stress and hypertensive heart disease [41–43]. We thus examined phosphorylation of ASK1 (Thr-845) and found that it was significantly increased in the BO group (Control (*n* = 4) vs. BO (*n* = 4); 1.0 ± 0.3 vs. 2.6 ± 1.4, *P* < 0.05 vs. Control). This increase was significantly inhibited by vidarabine (BO (*n* = 4) vs. BO + vidarabine (*n* = 5); 2.6 ± 1.4 vs. 0.5 ± 0.2, *P* < 0.01 vs. BO) (Fig. 5f).

These data suggest that ROS production derived from BO-induced NOX4 upregulation activates ASK1, leading to p38 MAPK and cardiac remodeling and dysfunction.

### Vidarabine inhibits BO-induced necroptosis

Overproduction of ROS derived from NOX4 was recently demonstrated to cause myocardial apoptosis and necroptosis, leading to heart failure via activation of RIP3 in cardiac myocytes [44]. We therefore examined the amount of phospho-RIP3 (Thr-231/Ser-232) and found that it was significantly increased in the heart of BO mice (Control (*n* = 5) vs. BO (*n* = 4); 1.0 ± 0.3 vs. 5.4 ± 2.3 A.U., *P* < 0.001 vs. Control) (Fig. 6a). Again, this increase was significantly attenuated by vidarabine (BO (*n* = 4) vs. BO + vidarabine (*n* = 4); 5.4 ± 2.3 vs. 1.7 ± 0.5 A.U., *P* < 0.01 vs. BO) (Fig. 6a).

These data suggest that activation of AC5 might contribute, at least in part, to the upregulation of NOX4 and XO.

### Vidarabine inhibits BO-induced CaMKII phosphorylation

RIP3-mediated myocardial apoptosis and necroptosis were recently demonstrated to be dependent upon the activation of CaMKII [44]. We therefore examined the amount of phospho-CaMKII (Thr-286) in the heart of BO mice and found that it was significantly increased at 2 weeks after BO (Control (*n* = 4) vs. BO (*n* = 5); 1.0 ± 0.2 vs. 1.9 ± 0.2 A.U., *P* < 0.001 vs. Control). The increase was significantly attenuated by vidarabine (BO (*n* = 5) vs. BO + vidarabine (*n* = 4); 1.9 ± 0.2 vs. 1.2 ± 0.3, *P* < 0.01 vs. BO) (Fig. 6b).

These data suggest that BO treatment might activate NOX4/RIP3/CaMKII signaling via activation of AC5.

### Vidarabine inhibits BO-induced PLN phosphorylation

The elevation of diastolic sarcoplasmic reticulum Ca^2+^ leakage mediated by PLN phosphorylation contributes considerably to the pathogenesis of cardiac remodeling and dysfunction via ROS derived from NOX4 [45, 46]. We therefore examined the effect of BO on PLN phosphorylation at Thr-17 and Ser-16, which are phosphorylated by CaMKII and by protein kinase A (Fig. 6c, d).

Phospho-PLN (Thr-17) was significantly increased in cardiac muscle of BO mice (Control (*n* = 4) vs. BO (*n* = 5); 1.0 ± 0.2 vs. 2.6 ± 1.3 A.U., *P* < 0.05 vs. Control) (Fig. 6c). This increase was significantly attenuated by vidarabine (BO (*n* = 5) vs. BO + vidarabine (*n* = 5); 2.6 ± 1.3% vs. 0.7 ± 0.2 A.U., *P* < 0.01 vs. BO) (Fig. 6c).

Phospho-PLN (Ser-16) was also significantly increased in cardiac muscle of BO mice (Control (*n* = 4) vs. BO (*n* = 5); 1.0 ± 0.5 vs. 2.2 ± 0.8 A.U., *P* < 0.05 vs. Control). Again, the increase was significantly attenuated by vidarabine (BO (*n* = 5) vs. BO + vidarabine (*n* = 5); 2.2 ± 0.8 vs. 0.8 ± 0.1 A.U., *P* < 0.01 vs. BO) (Fig. 6d).

These data, together with the previous results, suggest that BO might increase PLN phosphorylation, leading to ROS-mediated elevation of diastolic sarcoplasmic reticulum Ca^2+^ leakage in cardiac myocytes.

## Discussion

Oral health is important for maintaining general health and is associated with physical activity, including the status of the cardiovascular system [47]. Extensive studies have shown that the link between oral health and cardiovascular disease (CVD) may be explained by chronic inflammation and repeated bacteremia from the oral cavity, as inflammation plays an important role in the pathogenesis of CVD [1]. However, periodontal status may not completely explain the oral health–CVD relationship, and other factors such as occlusal disharmony, might also contribute [48].

Occlusal disharmony due to BO treatment causes muscle dysfunction and susceptibility to muscle fatigue in masseter and suprahyoid muscles via accumulation of ROS in rats [49, 50]. In addition, occlusal-disharmony-induced ROS production in the oral cavity might cause not only local pathogenic disturbance, but also systemic diseases, including heart disease, in patients [51]. Importantly, we previously showed that cardiac fibrosis and myocyte apoptosis were significantly increased in cardiac muscle of BO mice, together with accumulation of ROS, leading to cardiac dysfunction and susceptibility to atrial fibrillation via activation of β-AR signaling [15, 16]. Here, we examined whether AC5, a major cardiac AC isoform, is a mediator of the β-AR signaling leading to occlusal-disharmony-induced cardiac dysfunction, because AC5 is known to be a major regulator of oxidative stress in the heart [52, 53].

AC transduces the signal generated by binding of a ligand, most commonly norepinephrine, to β-AR and Gsα, resulting in the conversion of ATP to cAMP [54]. There are nine major mammalian isoforms of AC, with AC6 being the major fetal cardiac AC isoform, and AC5, the major cardiac isoform in adults [9, 54, 55]. We showed that in AC5KO, the heart was protected against the stress of chronic pressure overload [30] and chronic catecholamine stimulation [12]. Interestingly, however, AC5 inhibition appears to have multiple effects. For example, AC5 gene knockout or pharmacological AC5 inhibition increases longevity in mice [14, 20].

Inhibition of AC activity by P-site inhibitors including vidarabine is poor when AC is not fully stimulated [19, 56]. We have previously demonstrated that inhibition of AC5 by acute and chronic treatment with vidarabine did not affect cardiac function at baseline, but improved the response to pathological stress such as chronic catecholamine stress and permanent coronary artery occlusion [19, 20]. In addition, inhibition of AC5 also protects against physical frailty, enhances exercise capacity, and protects against diabetes, obesity and diabetic cardiomyopathy [14, 20, 52].

Several epidemiological surveys have confirmed a positive relationship between oral health and physical frailty, including cardiovascular disease [1, 57]. Tooth loss could contribute to occlusal disharmony or impaired masticatory performance [58–60]. More recently, a relationship between occlusal disharmony and cardiovascular disease was demonstrated in a Japanese urban population [1, 61]. However, the mechanism remains poorly understood.

**Fig. 7:**
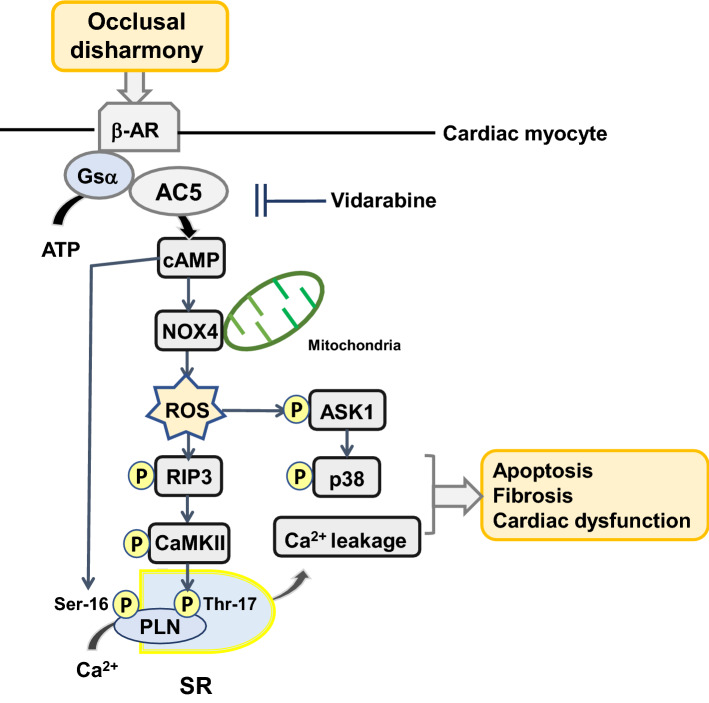
This scheme illustrates the proposed role of β-AR/Gsα/AC5 signaling in the heart of BO mice. β-AR/Gsα/AC5 signaling is activated by the BO treatment, leading to oxidative stress via activation of NOX4/ASK1/p38 and phosphorylation of CaMKII (Thr-286), which mediates PLN phosphorylation at Thr-17. In addition, cAMP derived from AC5 mediates oxidative stress and PLN phosphorylation at Ser-16. These changes might cause fibrosis, myocyte apoptosis and oxidative stress in the heart of BO mice, leading to cardiac dysfunction

In this study, pharmacological AC5 inhibition with vidarabine was shown to protect the heart from occlusal-disharmony-induced oxidative stress in BO mice, a well-established model of occlusal disharmony [15, 16, 24, 62]. In particular, our findings indicate that AC5 inhibition in BO mice has a cardioprotective effect mediated at least in part by a decrease of NOX4 overexpression and CaMKII phosphorylation, leading to reduced phospholamban phosphorylation on serine-16 and threonine-17 (Fig. 7).

NOX2 and NOX4 are the major isoforms of the NOX family enzymes in the heart, and both isoforms play an important role in mediating oxidative stress at baseline and under stress [35]. NOX2 is predominantly localized at the plasma membrane and NOX4 is predominantly localized in mitochondria [63, 64]. Therefore, upregulation of NOX4, but not NOX2, increases mitochondrial dysfunction, leading to apoptosis of cardiac myocytes and heart failure in response to pressure overload and chronic catecholamine stimulation [63, 65]. In addition, we demonstrated previously that disruption of the AC5 gene plays a protective role against the development of heart failure in response to pressure overload and chronic catecholamine stress, potentially through limiting the incidence of myocardial apoptosis [12, 30]. These data suggest that occlusal-disharmony-induced CVD might occur, at least in part, through the upregulation of NOX4 induced by activation of AC5 (Fig. 7).

We previously identified vidarabine as a candidate AC5 inhibitor by computer-based drug screening, and its inhibitory activity was confirmed in an in vitro cAMP accumulation assay in cardiac myocytes and also in AC assay using a crude membrane preparation [11, 19, 20]. The major finding of the current investigation is that inhibition of AC5 by vidarabine ameliorates the development of occlusal-disharmony-induced cardiac dysfunction by reducing the oxidative stress generated via the RIP3/NOX4 signaling pathway in a cAMP-dependent mechanism (Fig. 7). We have previously reported that vidarabine inhibits the development of catecholamine-induced heart failure and atrial fibrillation in mice without suppressing cardiac function [20, 66].

In previous studies, we demonstrated that vidarabine inhibits AC5 more potently than AC2 or AC3, which are more abundant in the lungs and pancreas, respectively [11]. Since β-AR is expressed in the pulmonary bronchus and pancreas, β-AR blockers cause bronchial smooth muscle contraction and inhibition of insulin release, leading to bronchospasm and glucose intolerance [67, 68]. Our current findings suggest that the use of vidarabine to suppress only the activity of AC5, and not the entire β-AR signaling pathway, may be preferable to β-AR blockade therapy for the treatment of CVD associated with occlusal-disharmony.

## Conclusion

Our current and previous findings suggest that vidarabine might broadly inhibit stress-induced cardiomyopathy, leading to improved longevity and reduced physical frailty. Since vidarabine is a clinically approved drug, an early clinically trial should be feasible.

### Supplementary Information

**Additional file 1: Figure S1.** The original photo of Fig. 1b. The box outlined in black, indicated by the arrow, corresponds to the cropped part shown in the main article. **Figure S2.** Food consumption and (a) and drinking water (b) throughout the 2-week experimental period. C: Control, B: BO, V: Vidarabine, BO + V: BO + Vidarabine.** Fig. S3.** Representative full-length western blotting of Fig. 2c. The amounts of activated α-SMA (left panel) and GAPDH (right panel) are shown. The box outlined in black in each panel, indicated by the arrow, corresponds to the cropped part of the blot shown in the main article. C: Control, B: BO, V: Vidarabine, BOV: BO + Vidarabine. **Fig. S4.** Representative full-length western blotting of Fig. 3c. The amounts of Bax (left panel) and BCL-2 (right panel) are shown. The box outlined in black in each panel, indicated by the arrow, corresponds to the cropped parts of the blot shown in the main article. C: Control, B: BO, V: Vidarabine, BOV: BO + Vidarabine. **Fig. S5.** Representative images of negative (left) and positive (right) controls of 8-OHdG immunostaining. **Fig. S6.** Representative full-length western blotting of Fig. 4c. The amounts of activated oxidized protein (left panel) and GAPDH (right panel) are shown. The box outlined in black in each panel, indicated by the arrow, corresponds to the cropped part of the blot. C: control, B: BO, V: Vidarabine, BOV: BO + Vidarabine. **Fig. S7.** Representative full-length western blotting of Fig. 5a. The amounts of AC5 (left panel) and GAPDH (right panel) are shown. The box outlined in black in each panel, indicated by the arrow, corresponds to the cropped part of the blot shown in the main article. C: Control, B: BO, V: Vidarabine, BOV: BO + Vidarabine. **Fig. S8.** Representative full-length western blotting of Fig. 5b. The amounts of NOX4 (left panel) and GAPDH (right panel) are shown. The box outlined in black in each panel, indicated by the arrow, corresponds to the cropped part of the blot shown in the main article. C: Control, B: BO, V: Vidarabine, BOV: BO + Vidarabine. **Fig. S9.** Representative full-length western blotting of Fig. 5c. The amounts of NOX2 (left panel) and GAPDH (right panel) are shown. The box outlined in black in each panel, indicated by the arrow, corresponds to the cropped part of the blot shown in the main article. C: Control, B: BO, V: Vidarabine, BOV: BO + Vidarabine. **Fig. S10.** Representative full-length western blotting of Fig. 5d. The amounts of XO (left panel) and GAPDH (right panel) are shown. The box outlined in black in each panel, indicated by the arrow, corresponds to the cropped part of the blot shown in the main article. C: Control, B: BO, V: Vidarabine, BOV: BO + Vidarabine. **Fig. S11.** Representative full-length western blotting of Fig. 5e. The amounts of phosphorylated p38 at Thr-180/Tyr-182 (left panel) and total p38 (right panel) are shown. The box outlined in black in each panel, indicated by the arrow, corresponds to the cropped part of the blot shown in the main article. C: Control, B: BO, V: Vidarabine, BOV: BO + Vidarabine. **Fig. S12.** Representative full-length western blotting of Fig. 5f. The amounts of phosphorylated ASK-1 at Thr-845 (left panel) and total ASK-1 (right panel) are shown. The box outlined in black in each panel, indicated by the arrow, corresponds to the cropped part of the blot shown in the main article. C: control, B: BO, V: Vidarabine, BOV: BO + Vidarabine. **Fig. S13.** Representative full-length western blotting of Fig. 6a. The amounts of RIP3 (left panel) and GAPDH (right panel) are shown. The box outlined in black in each panel, indicated by the arrow, corresponds to the cropped part of the blot shown in the main article. C: control, B: BO, V: Vidarabine, BOV: BO + Vidarabine. **Fig. S14.** Representative full-length western blotting of Fig. 6b. The amounts of phosphorylated CaMKII at Thr-286 (left panel) and total CaMKII (right panel) are shown. The box outlined in black in each panel, indicated by the arrow, corresponds to the cropped part of the blot shown in the main article. C: Control, B: BO, V: Vidarabine, BOV: BO + Vidarabine. **Fig. S15.** Representative full-length western blotting of Fig. 6c. The amounts of phosphorylated PLB at Thr-17 (left panel) and total PLB (right panel) are shown. The box outlined in black in each panel, indicated by the arrow, corresponds to the cropped part of the blot shown in the main article. C: Control, B: BO, V: Vidarabine, BOV: BO + Vidarabine. **Fig. S16.** Representative full-length western blotting of Fig. 6d. The amounts of phosphorylated PLB at Ser-16 (left panel) and total PLB (right panel) are shown. The box outlined in black in each panel, indicated by the arrow, corresponds to the cropped part of the blot shown in the main article. C: Control, B: BO, V: Vidarabine, BOV: BO + Vidarabine.

## Data Availability

The datasets used and/or analyzed during the current study are available from the corresponding author on reasonable request.

## Abbreviations

AC5: Type 5 adenylyl cyclase
BO: Bite-opening
AC: Adenylyl cyclase
AC5KO: Mouse model with knock-out of AC5
BO: Bite-opening
β-AR: β-Adrenergic receptor
BW: Body weight
TUNEL: Terminal deoxyribonucleotidyl transferase (TdT)-mediated biotin-16-deoxyuridine
α-SMA: α-Smooth muscle actin
CaMKII: Calmodulin kinase II
BCL-2: B cell lymphoma 2
Bax: BCL-2 associated X
RIP3: Receptor-interacting protein 3
ASK1: Apoptosis signal-regulatory kinase 1
GAPDH: Glyceraldehyde-3-phosphate dehydrogenase
PLN: Phospholamban
NOX: Nicotinamide adenine dinucleotide phosphate oxidase
XO: Xanthine oxidase
8-OHdG: 8-Hydroxy-2’-deoxyguanosine
A.U.: Arbitrary unit
EF: Left ventricular cardiac function
%FS: %Fractional shortening
ROS: Reactive oxygen species
MAPK: Mitogen-activated protein kinase
CVD: Cardiovascular disease
