# Supplementary material for: Vidarabine, an anti-herpes agent, prevents occlusal-disharmony-induced cardiac dysfunction in mice
Source: J Physiol Sci. 2022 Feb 11;72:2. doi: 10.1186/s12576-022-00826-4 (PMC10717220; doi:10.1186/s12576-022-00826-4)
Supplement: Supplementary file 1 — Additional file 1: Figure S1. The original photo of Fig. 1b. The box outlined in black, indicated by the arrow, corresponds to the cropped part shown in the main article. Figure S2. Food consumption and (a) and drinking water (b) throughout the 2-week experimental period. C: Control, B: BO, V: Vidarabine, BO + V: BO + Vidarabine. Fig. S3. Representative full-length western blotting of Fig. 2c. The amounts of activated α-SMA (left panel) and GAPDH (right panel) are shown. The box outlined in black in each panel, indicated by the arrow, corresponds to the cropped part of the blot shown in the main article. C: Control, B: BO, V: Vidarabine, BOV: BO + Vidarabine. Fig. S4. Representative full-length western blotting of Fig. 3c. The amounts of Bax (left panel) and BCL-2 (right panel) are shown. The box outlined in black in each panel, indicated by the arrow, corresponds to the cropped parts of the blot shown in the main article. C: Control, B: BO, V: Vidarabine, BOV: BO + Vidarabine. Fig. S5. Representative images of negative (left) and positive (right) controls of 8-OHdG immunostaining. Fig. S6. Representative full-length western blotting of Fig. 4c. The amounts of activated oxidized protein (left panel) and GAPDH (right panel) are shown. The box outlined in black in each panel, indicated by the arrow, corresponds to the cropped part of the blot. C: control, B: BO, V: Vidarabine, BOV: BO + Vidarabine. Fig. S7. Representative full-length western blotting of Fig. 5a. The amounts of AC5 (left panel) and GAPDH (right panel) are shown. The box outlined in black in each panel, indicated by the arrow, corresponds to the cropped part of the blot shown in the main article. C: Control, B: BO, V: Vidarabine, BOV: BO + Vidarabine. Fig. S8. Representative full-length western blotting of Fig. 5b. The amounts of NOX4 (left panel) and GAPDH (right panel) are shown. The box outlined in black in each panel, indicated by the arrow, corresponds to the cropped part of the blot shown in the m [file 12576_2022_826_MOESM1_ESM.pdf]

Additional File 1

Vidarabine, an anti-herpes agent, prevents occlusal-disharmony-induced cardiac dysfunction in mice

Yoshio Hayakawa [1,2], Kenji Suita [1], Yoshiki Ohnuki [1], Yasumasa Mototani [1], Misao Ishikawa [3], Aiko Ito [4], Megumi Nariyama [5], Akinaka Morii [1,6], Kenichi Kiyomoto [1,6], Michinori Tsunoda [1,6], Ichiro Matsuo [1,6], Hiroshi Kawahara [2], Satoshi Okumura [1]

[1] Department of Physiology, Tsurumi University School of Dental Medicine, Yokohama 230-8501, Japan

[2] Department of Dental Anesthesiology, Tsurumi University School of Dental Medicine, Yokohama 230-8501, Japan

[3] Department of Oral Anatomy, Tsurumi University School of Dental Medicine, Yokohama 230-8501, Japan

[4] Department of Orthodontics, Tsurumi University School of Dental Medicine, Yokohama 230-8501, Japan

[5] Department of Pediatric Dentistry, Tsurumi University School of Dental Medicine, Yokohama 236-8501, Japan

[6] Department of Periodontology, Tsurumi University School of Dental Medicine, Yokohama 230-8501, Japan

***Corresponding author: Satoshi Okumura:

Department of Physiology, Tsurumi University School of Dental Medicine,

2-1-3 Tsurumi, Tsurumi-ku, Yokohama 230-8501; (Tel. +81-(0)45-580-8476;

Fax. +81-(0)45-585-2889; e-mail: okumura-s@tsurumi-u.ac.jp)

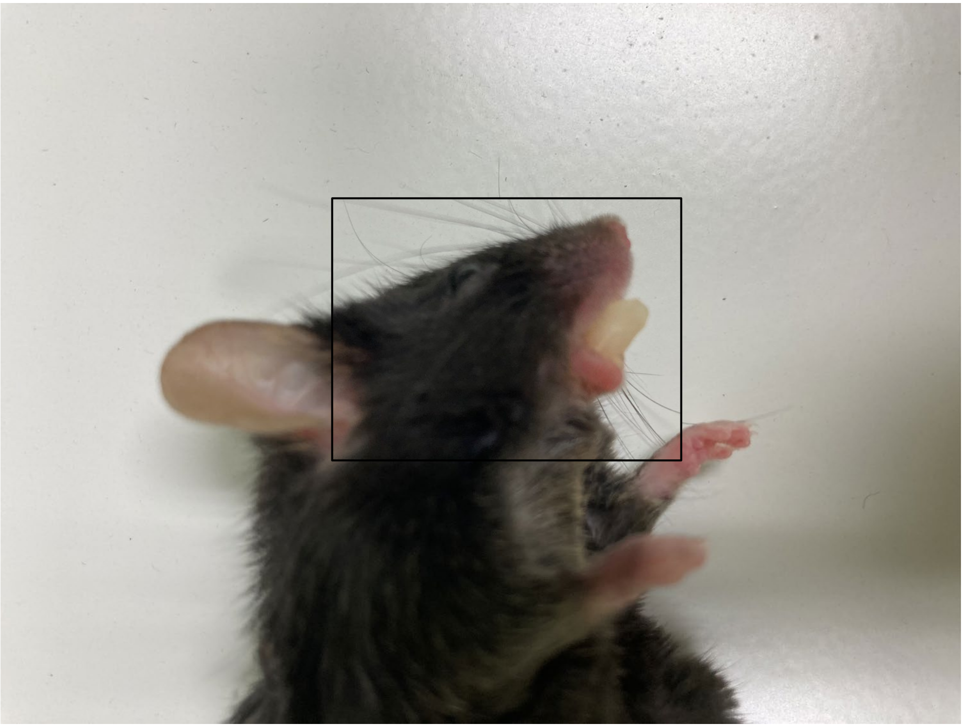

Fig. S1
The original photo of **Fig. 1b**. The box outlined in black, indicated by the arrow, corresponds to the cropped part shown in the main article.

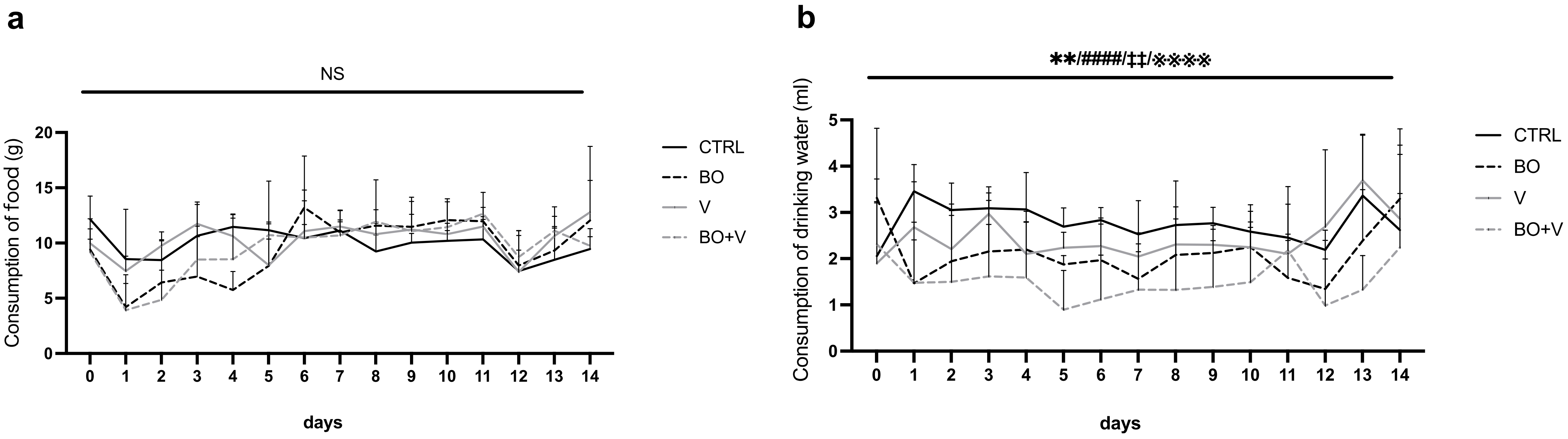

CTRL vs. BO : ＊＊ (*P* < 0.01)
CTRL vs. BO + V : ＃ ＃ ＃ ＃(*P* < 0.0001)
BO vs. V : ‡ ‡ : (*P* < 0.01)
V vs. BO + V : ※ ※ ※ ※(*P* < 0.0001)
ns: not significantly different
(two-way repeated-measures ANOVA by the Bonferroni *post hoc* test)

Fig. S2

Food consumption and **(a)** and drinking water **(b)** throughout the 2-week experimental period. C: Control, B: BO, V: Vidarabine, BO + V: BO + Vidarabine

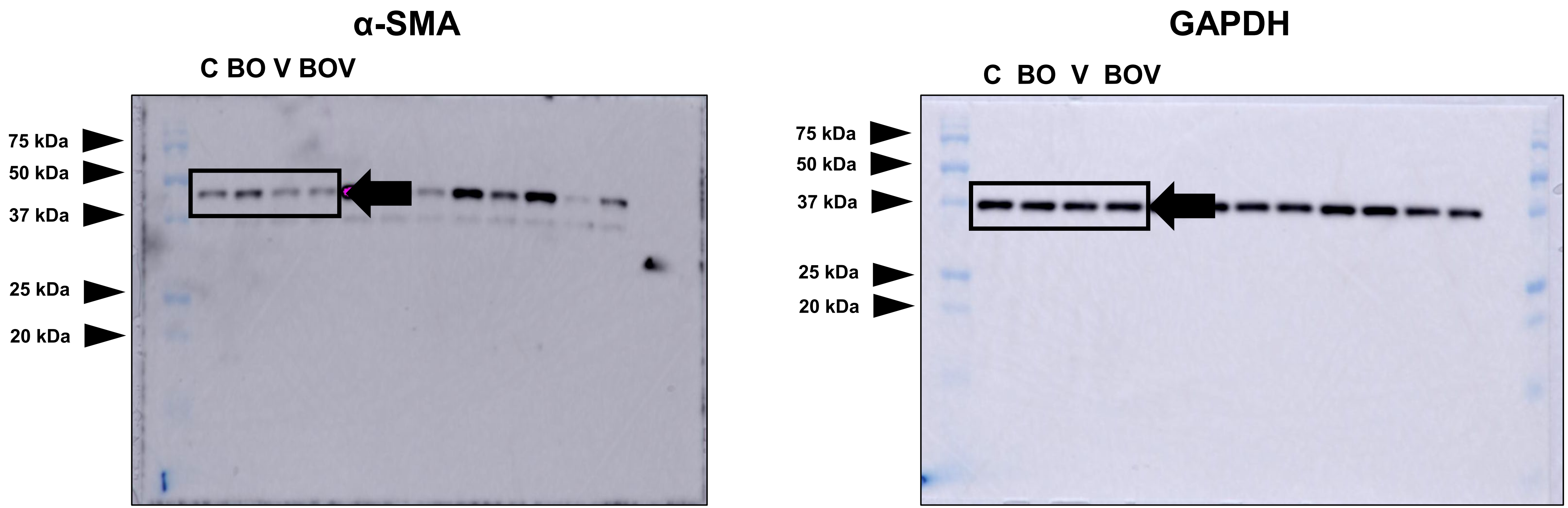

Fig. S3
Representative full-length western blotting of **Fig. 2c**. The amounts of activated α-SMA (left panel) and GAPDH (right panel) are shown. The box outlined in black in each panel, indicated by the arrow, corresponds to the cropped part of the blot shown in the main article. C: Control, B: BO, V: Vidarabine, BOV: BO + Vidarabine

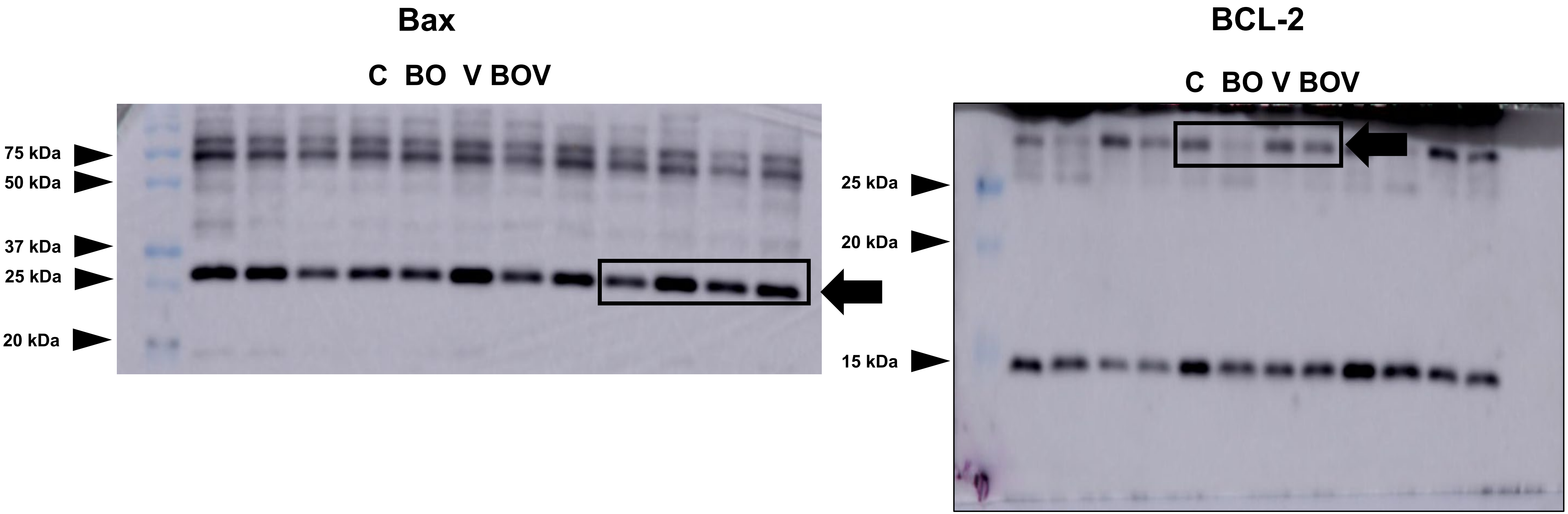

Fig. S4

Representative full-length western blotting of **Fig. 3c**. The amounts of Bax (left panel) and BCL-2 (right panel) are shown. The box outlined in black in each panel, indicated by the arrow, corresponds to the cropped parts of the blot shown in the main article. C: Control, B: BO, V: Vidarabine, BOV: BO + Vidarabine

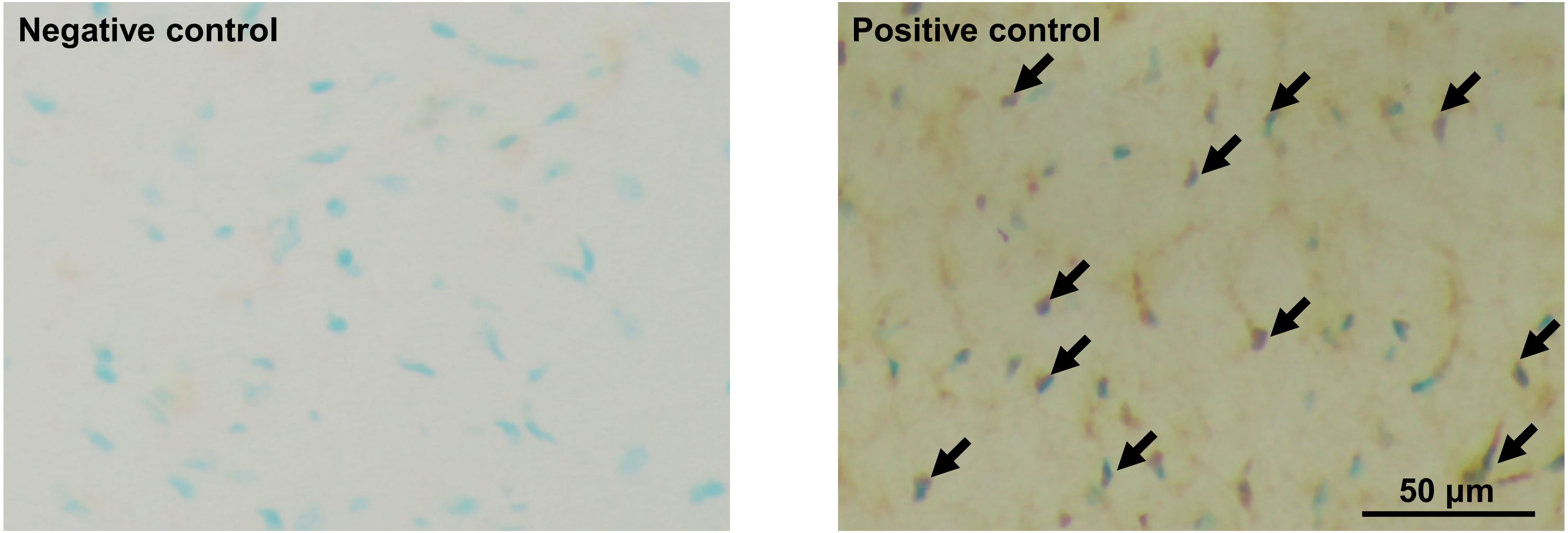

Fig. S5

Representative images of negative (left) and positive (right) controls of 8-OHdG immunostaining.

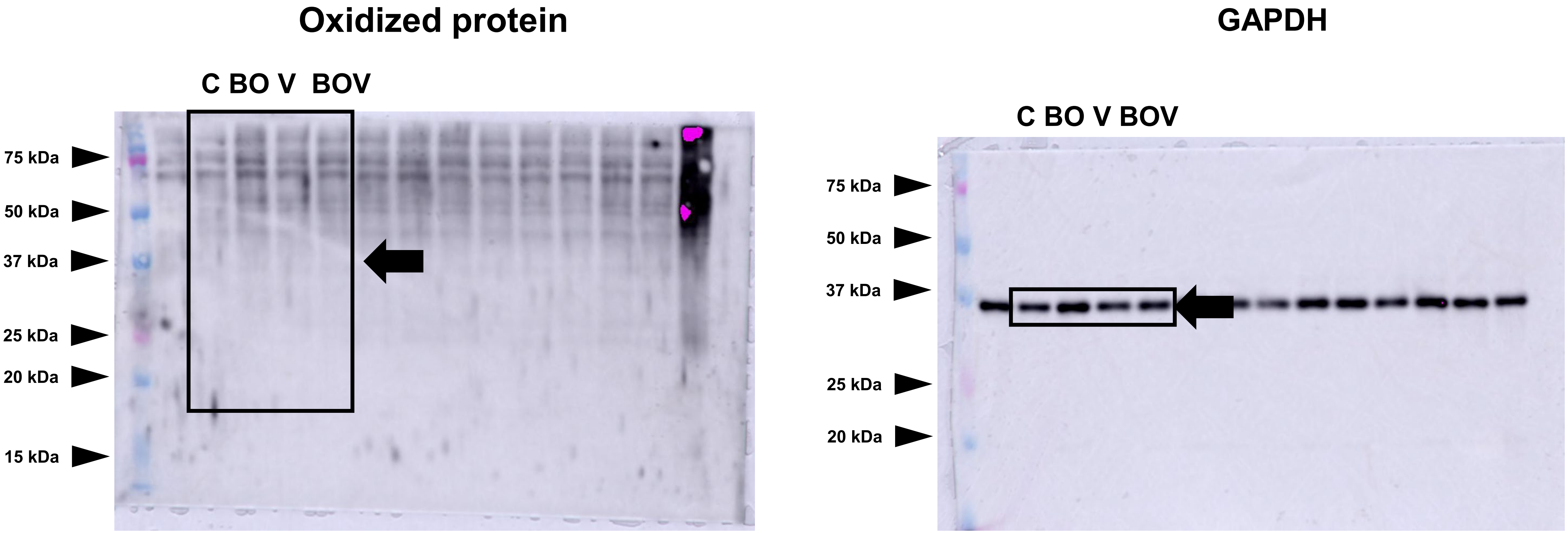

Fig. S6

Representative full-length western blotting of **Fig. 4c**. The amounts of activated oxidized protein (left panel) and GAPDH (right panel) are shown. The box outlined in black in each panel, indicated by the arrow, corresponds to the cropped part of the blot. C: control, B: BO, V: Vidarabine, BOV: BO + Vidarabine

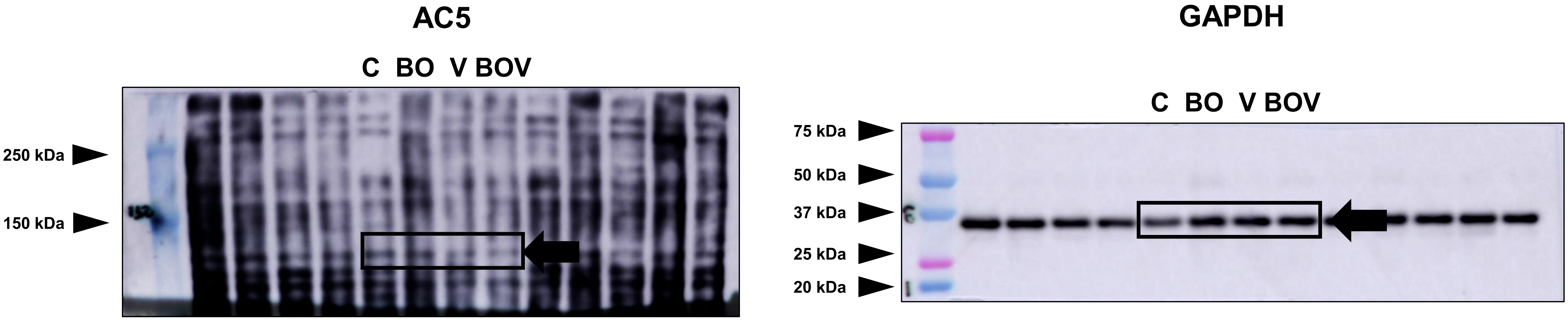

Fig. S7

Representative full-length western blotting of **Fig. 5a**. The amounts of AC5 (left panel) and GAPDH (right panel) are shown. The box outlined in black in each panel, indicated by the arrow, corresponds to the cropped part of the blot shown in the main article. C: Control, B: BO, V: Vidarabine, BOV: BO + Vidarabine

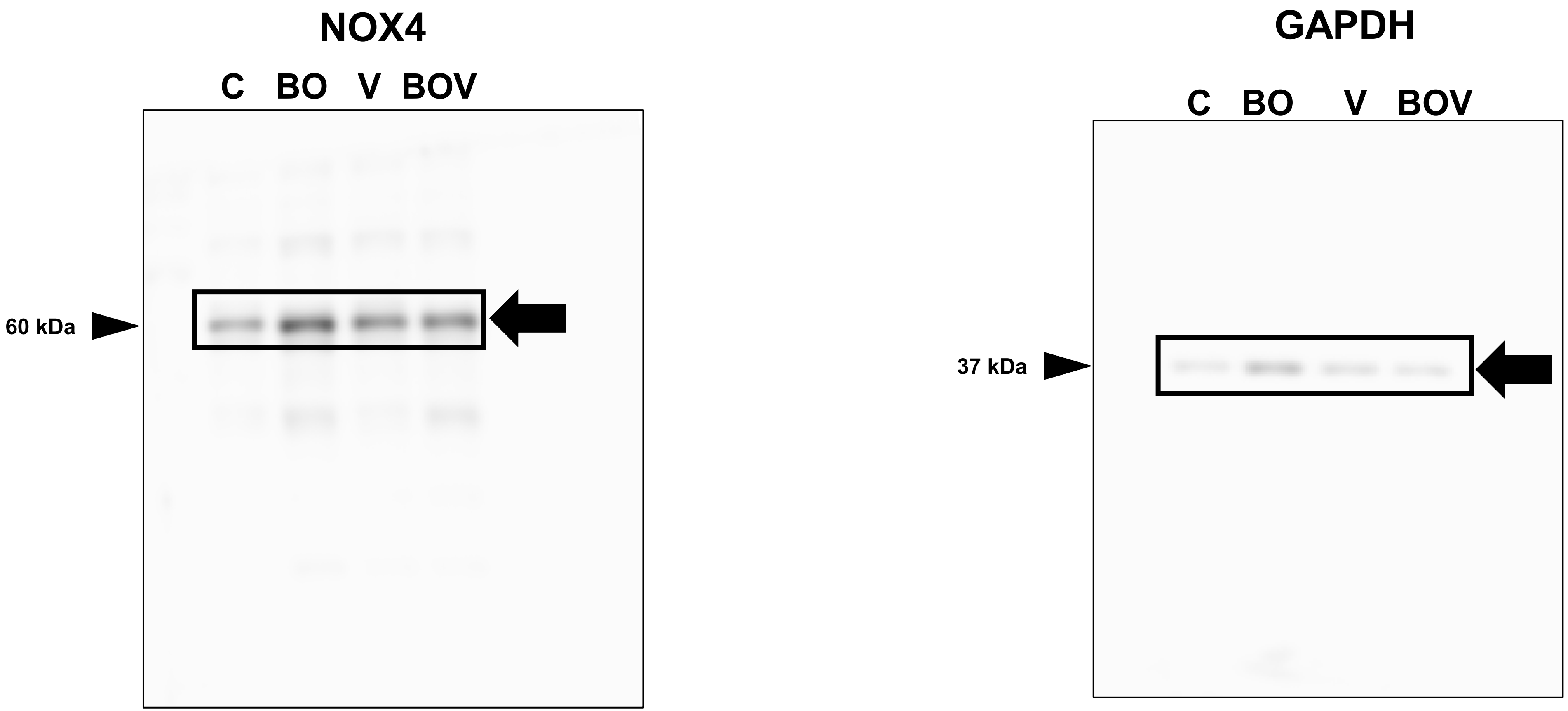

Fig. S8

Representative full-length western blotting of **Fig. 5b**. The amounts of NOX4 (left panel) and GAPDH (right panel) are shown. The box outlined in black in each panel, indicated by the arrow, corresponds to the cropped part of the blot shown in the main article. C: Control, B: BO, V: Vidarabine, BOV: BO + Vidarabine

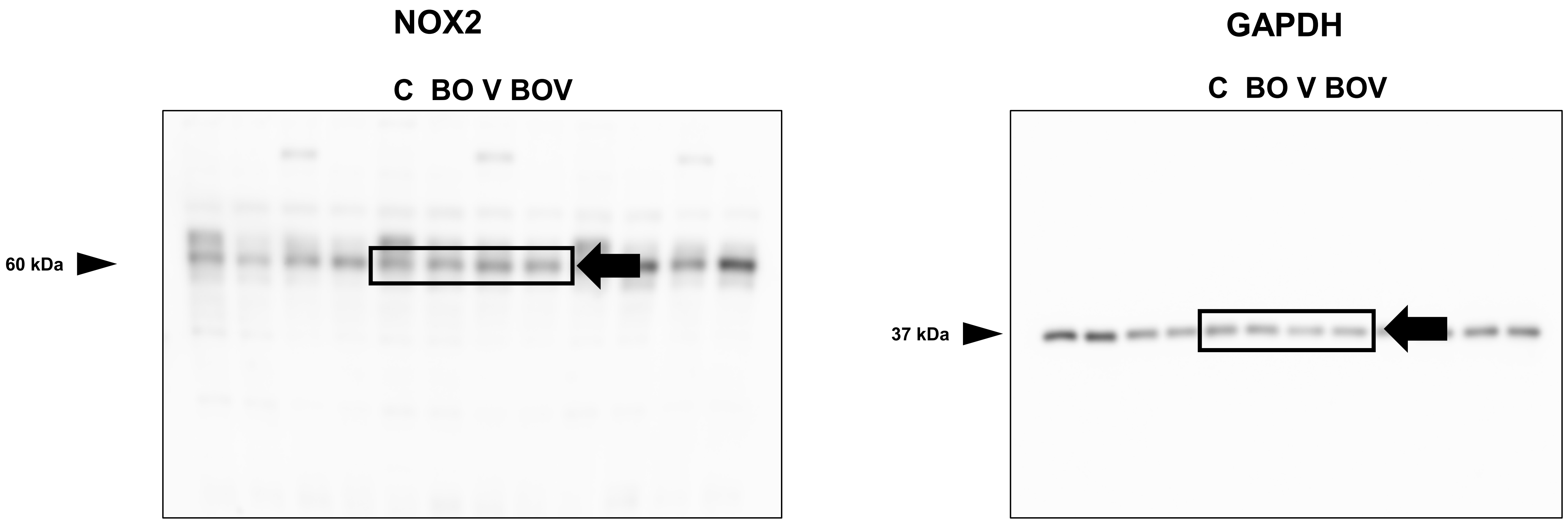

Fig. S9

Representative full-length western blotting of **Fig. 5c**. The amounts of NOX2 (left panel) and GAPDH (right panel) are shown. The box outlined in black in each panel, indicated by the arrow, corresponds to the cropped part of the blot shown in the main article. C: Control, B: BO, V: Vidarabine, BOV: BO + Vidarabine

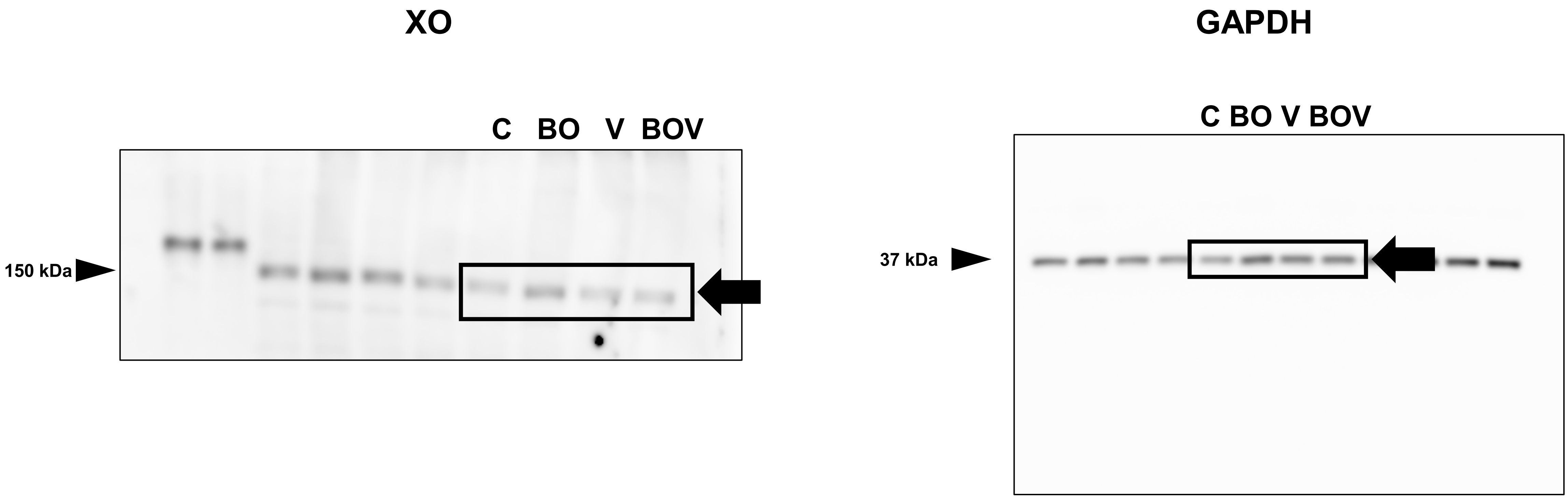

Fig. S10

Representative full-length western blotting of **Fig. 5d**. The amounts of XO (left panel) and GAPDH (right panel) are shown. The box outlined in black in each panel, indicated by the arrow, corresponds to the cropped part of the blot shown in the main article. C: Control, B: BO, V: Vidarabine, BOV: BO + Vidarabine

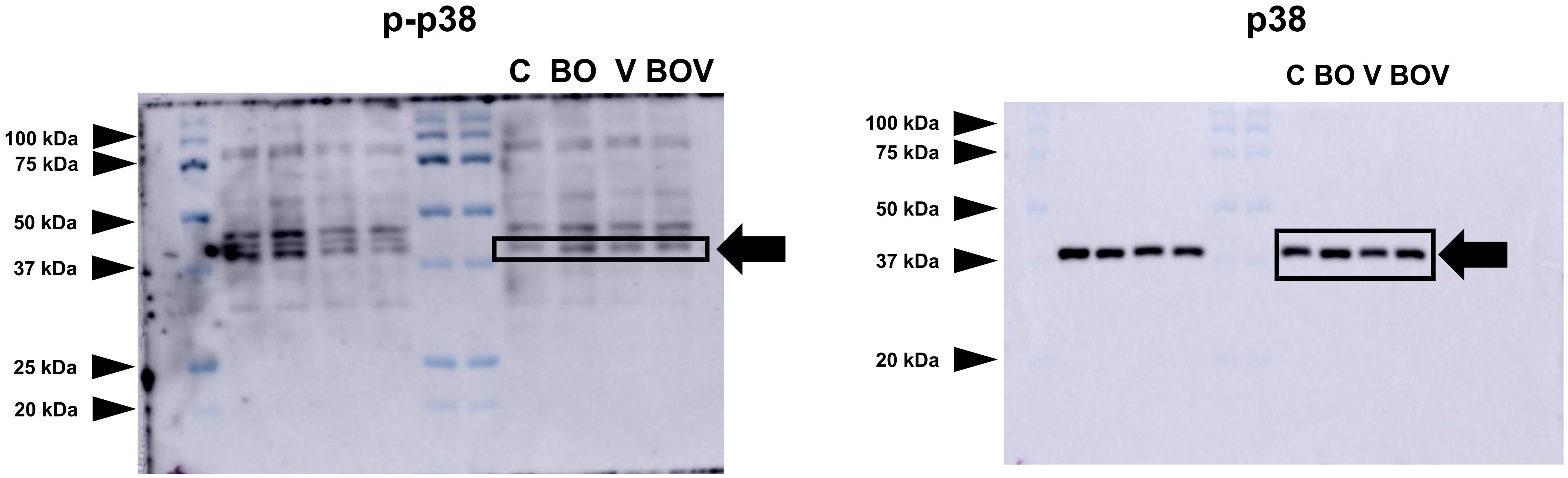

Fig. S11

Representative full-length western blotting of **Fig. 5e**. The amounts of phosphorylated p38 at Thr-180/Tyr-182 (left panel) and total p38 (right panel) are shown. The box outlined in black in each panel, indicated by the arrow, corresponds to the cropped part of the blot shown in the main article. C: Control, B: BO, V: Vidarabine, BOV: BO + Vidarabine

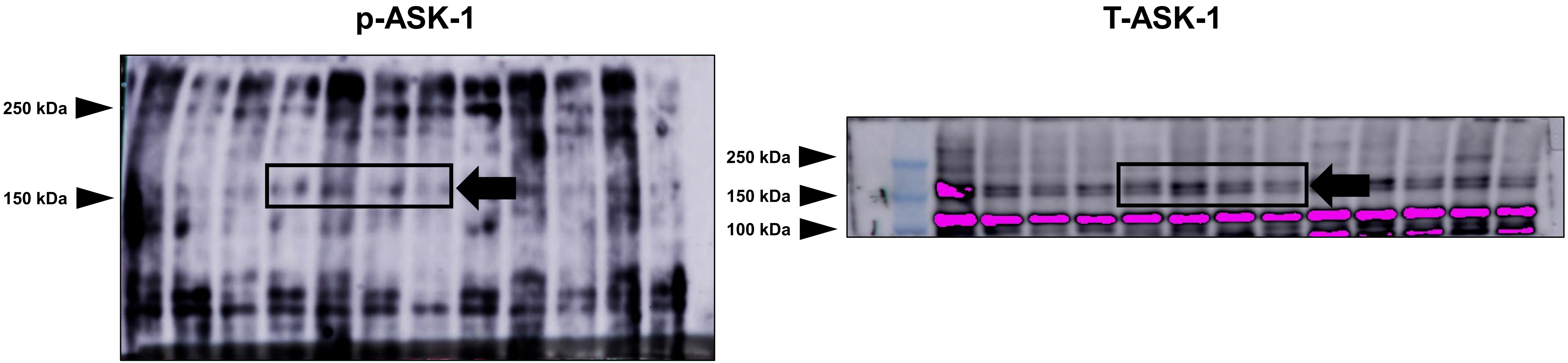

Fig. S12

Representative full-length western blotting of **Fig. 5f**. The amounts of phosphorylated ASK-1 at Thr-845 (left panel) and total ASK-1 (right panel) are shown. The box outlined in black in each panel, indicated by the arrow, corresponds to the cropped part of the blot shown in the main article. C: control, B: BO, V: Vidarabine, BOV: BO + Vidarabine

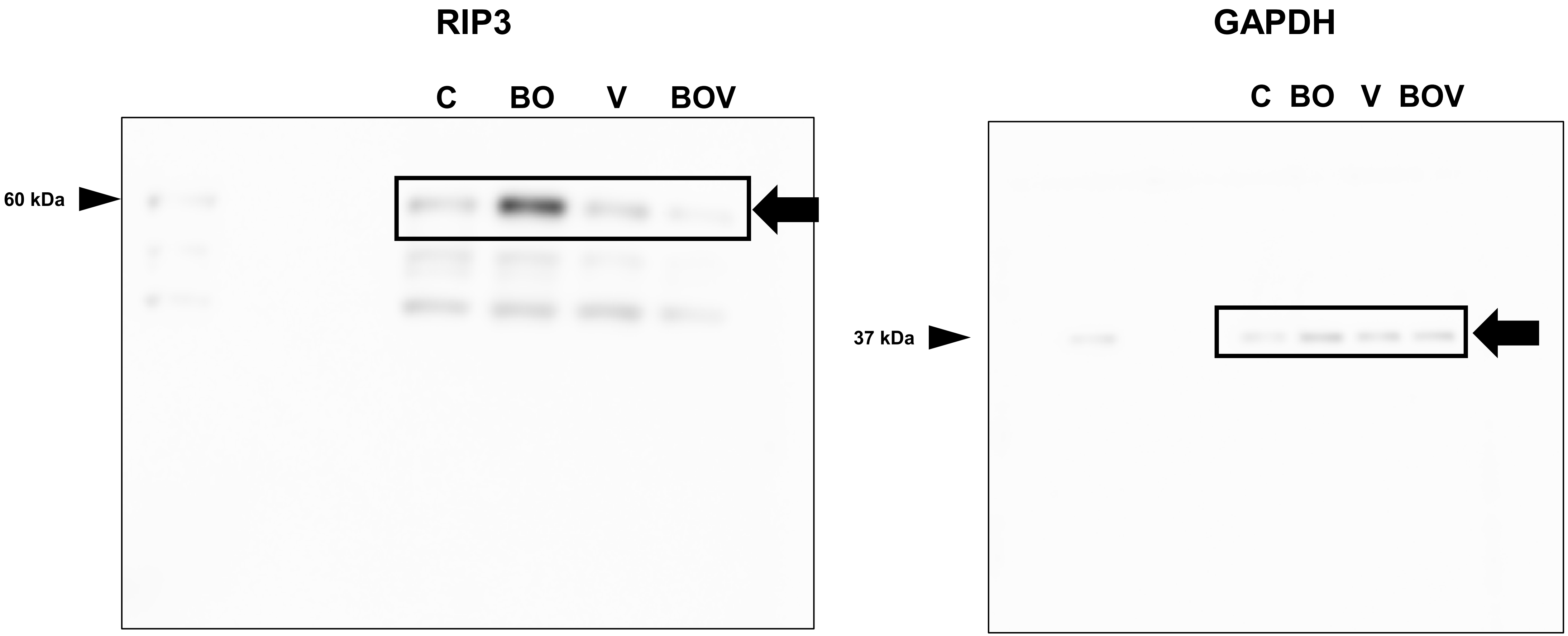

Fig. S13

Representative full-length western blotting of **Fig. 6a**. The amounts of RIP3 (left panel) and GAPDH (right panel) are shown. The box outlined in black in each panel, indicated by the arrow, corresponds to the cropped part of the blot shown in the main article. C: control, B: BO, V: Vidarabine, BOV: BO + Vidarabine

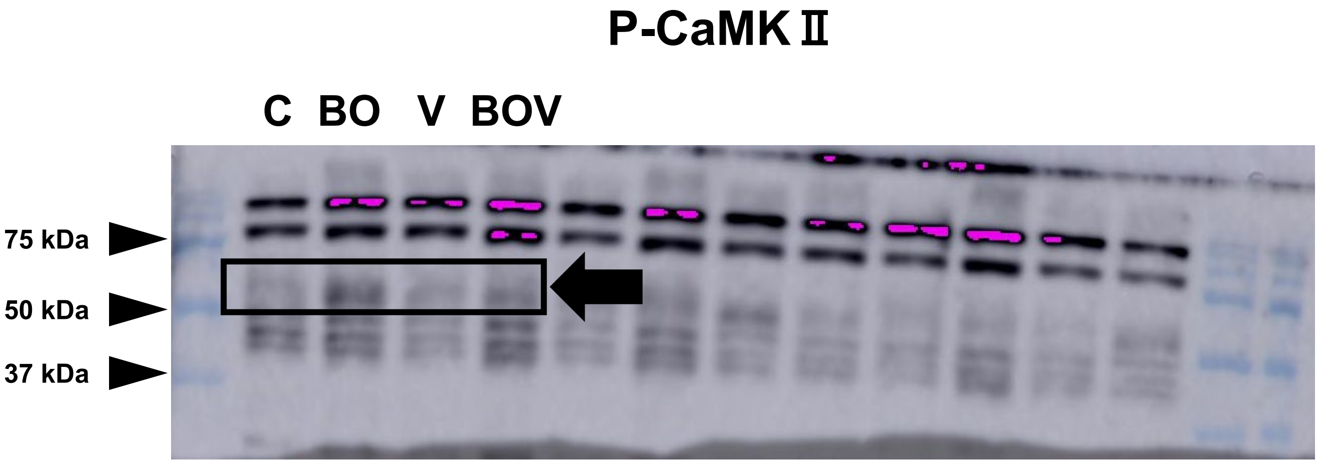

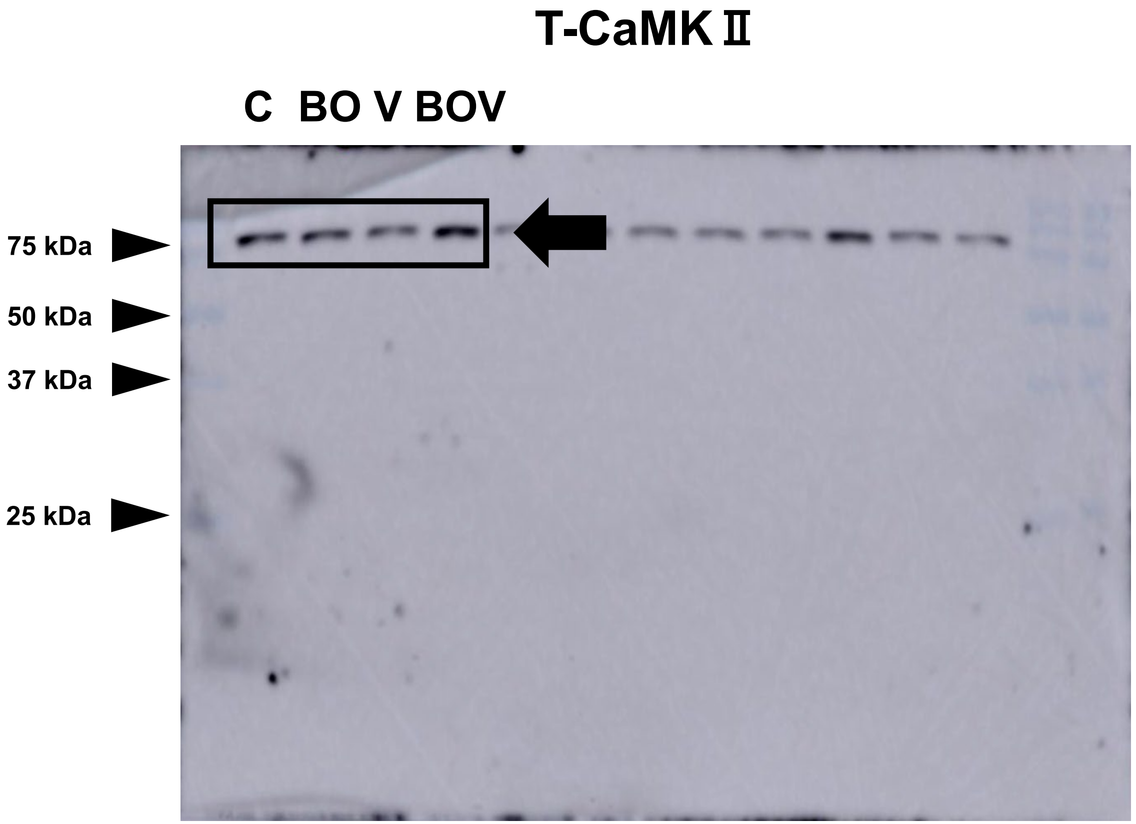

Fig. S14

Representative full-length western blotting of **Fig. 6b**. The amounts of phosphorylated CaMKII at Thr-286 (left panel) and total CaMKII (right panel) are shown. The box outlined in black in each panel, indicated by the arrow, corresponds to the cropped part of the blot shown in the main article. C: Control, B: BO, V: Vidarabine, BOV: BO + Vidarabine

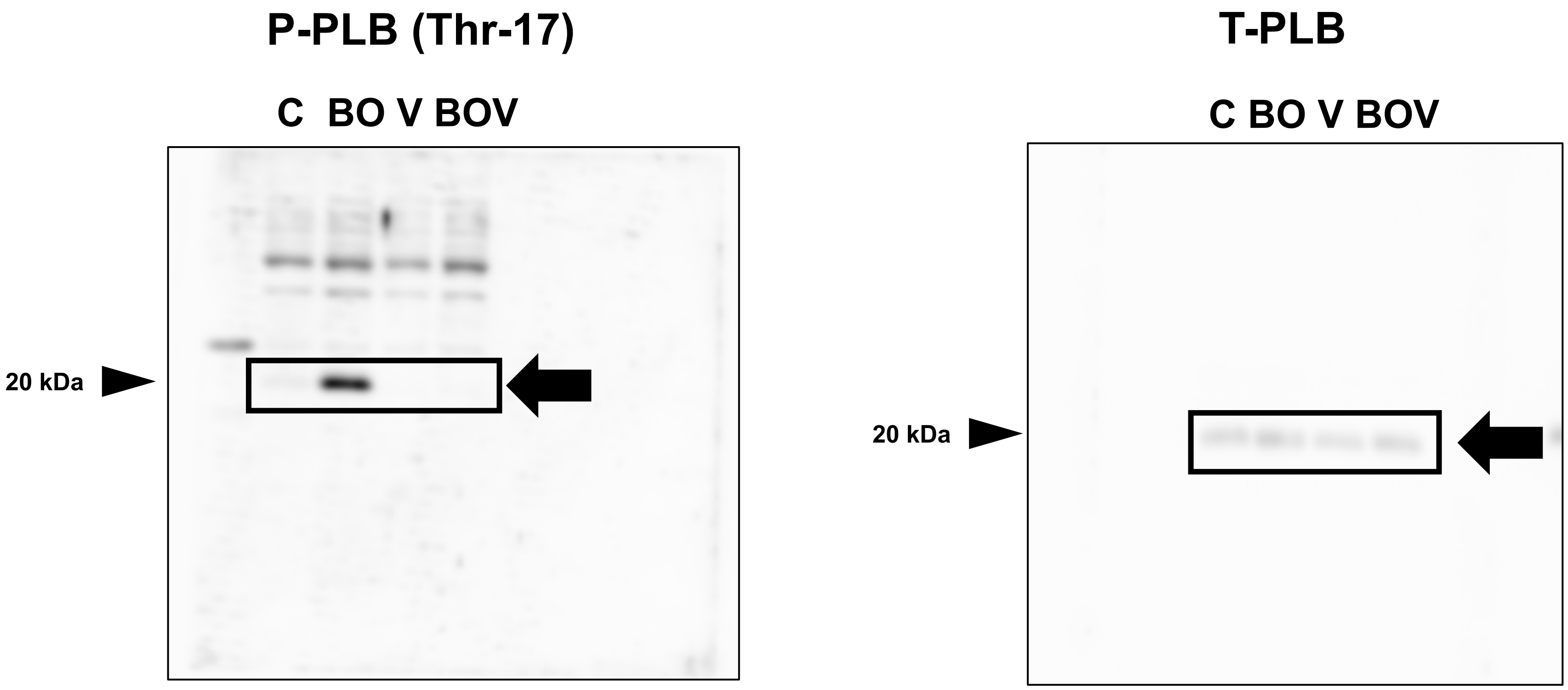

Fig. S15
Representative full-length western blotting of **Fig. 6c**. The amounts of phosphorylated PLB at Thr-17 (left panel) and total PLB (right panel) are shown. The box outlined in black in each panel, indicated by the arrow, corresponds to the cropped part of the blot shown in the main article. C: Control, B: BO, V: Vidarabine, BOV: BO + Vidarabine

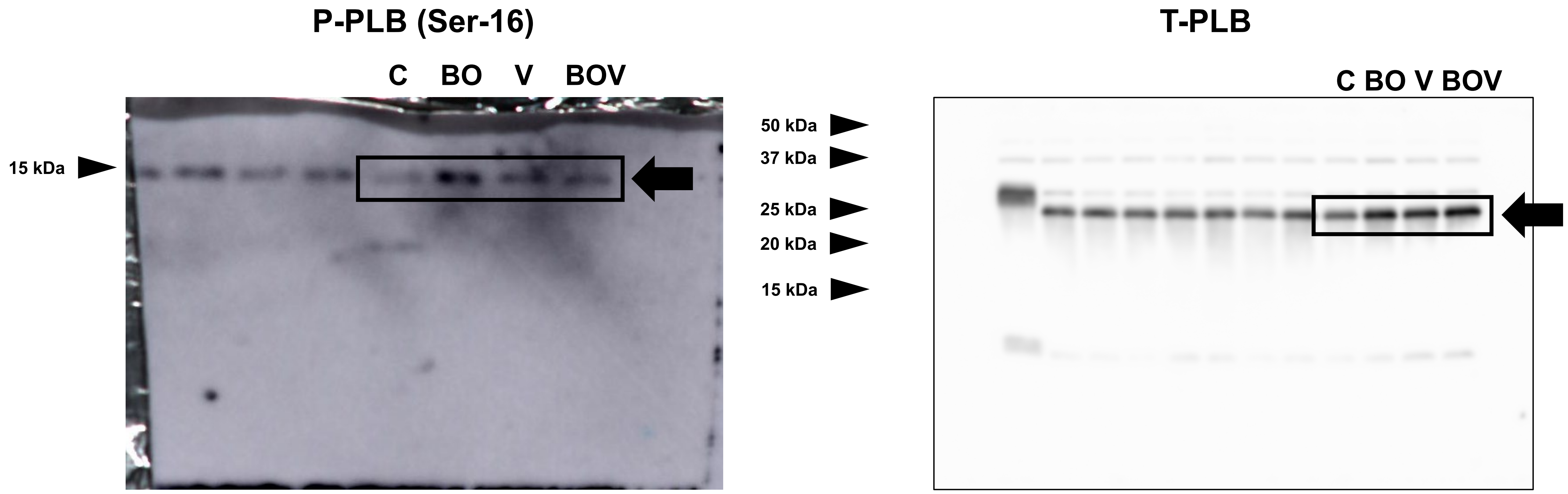

Fig. S16

Representative full-length western blotting of **Fig. 6d**. The amounts of phosphorylated PLB at Ser-16 (left panel) and total PLB (right panel) are shown. The box outlined in black in each panel, indicated by the arrow, corresponds to the cropped part of the blot shown in the main article. C: Control, B: BO, V: Vidarabine, BOV: BO + Vidarabine
